# Staged Models for Interdisciplinary Research

**DOI:** 10.1371/journal.pone.0157261

**Published:** 2016-06-30

**Authors:** Luis F. Lafuerza, Louise Dyson, Bruce Edmonds, Alan J. McKane

**Affiliations:** 1 Theoretical Physics, School of Physics and Astronomy, University of Manchester, Manchester M13 9PL, United Kingdom; 2 Centre for Policy Modelling, Manchester Metropolitan University, Manchester, M15 6BH, United Kingdom; Cinvestav-Merida, MEXICO

## Abstract

Modellers of complex biological or social systems are often faced with an invidious choice: to use simple models with few mechanisms that can be fully analysed, or to construct complicated models that include all the features which are thought relevant. The former ensures rigour, the latter relevance. We discuss a method that combines these two approaches, beginning with a complex model and then modelling the complicated model with simpler models. The resulting “chain” of models ensures some rigour and relevance. We illustrate this process on a complex model of voting intentions, constructing a reduced model which agrees well with the predictions of the full model. Experiments with variations of the simpler model yield additional insights which are hidden by the complexity of the full model. This approach facilitated collaboration between social scientists and physicists—the complex model was specified based on the social science literature, and the simpler model constrained to agree (in core aspects) with the complicated model.

## Introduction

To a surprising degree, the physical world can be understood through simple models (where by ‘simple models’ we mean those that can be fully analysed). However, it is inevitable that some phenomena will not be adequately represented in this way, as seems to be the case for many biological and social systems [1]. In such cases, the scientist is faced with an invidious choice: to use a simple model that can be rigorously understood but does not adequately capture the phenomena of interest; or to use a complex model that includes all the details considered necessary, but may be impossible to analyse. When trying to understand very complex phenomena, researchers from different disciplines have tended to prioritise different goals in modelling, theoretical physicists emphasizing analytical tractability and social scientists concentrating more on relevance.

**Fig 1:**
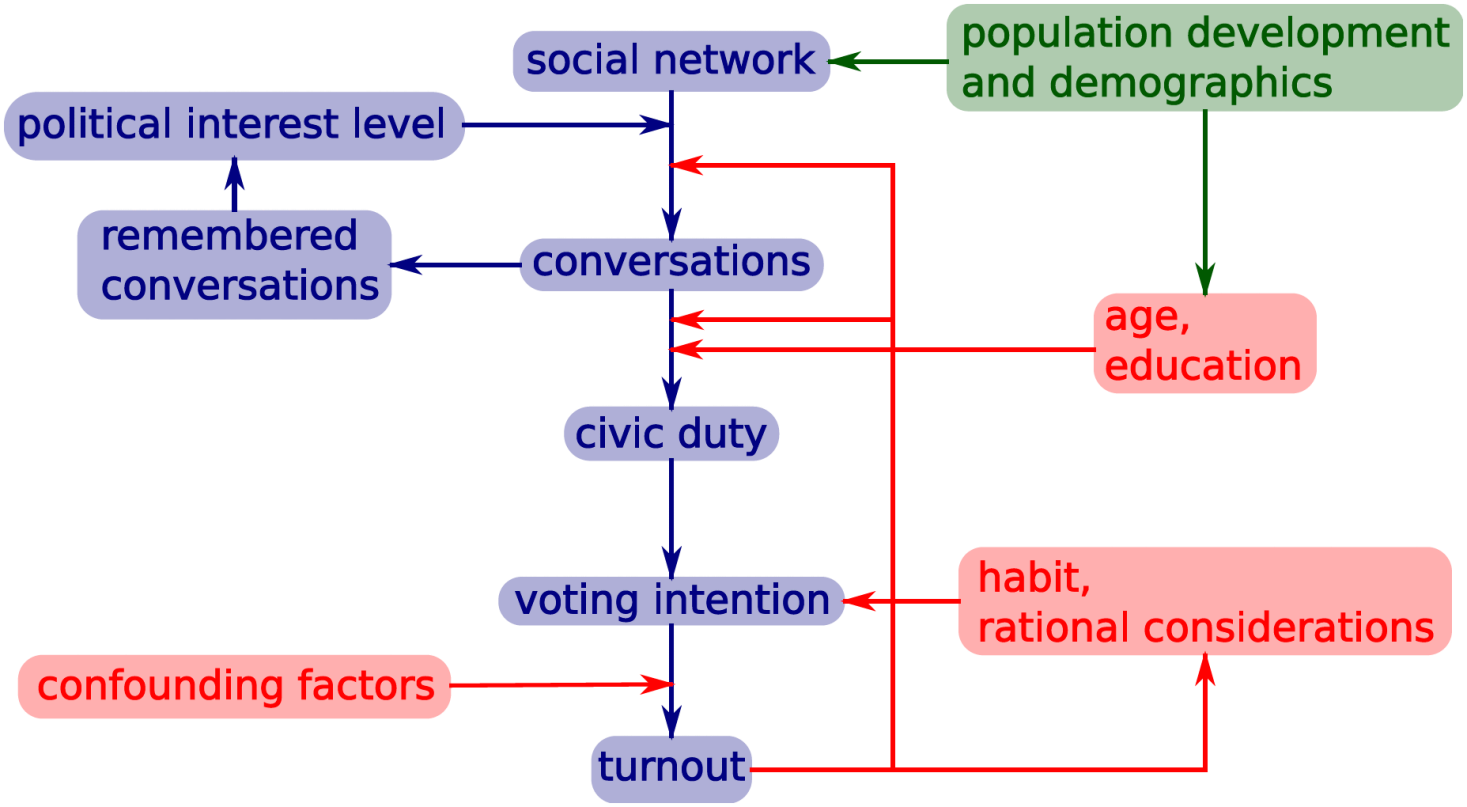
From a single to a multi-stage abstraction process.

In this paper we suggest and demonstrate a method which attempts to combine some of the best features of both approaches. This method stages the modelling process by constructing a “chain” of models, instead of jumping to a relatively simple model immediately (Fig 1). It starts with a complex but incompletely understood model and then reduces it to a simpler model that approximates some behaviours of the original. By using two, closely related, models rather than one, we hope to (a) ground the relevance of the specification of the simpler model; (b) identify key behaviours that are amenable to relatively simple representation; (c) understand the conditions under which this simplification may hold; and (d) better understand the more complex model. The disadvantages of the approach mostly relate to the increased work involved in construction and comparison.

However, such an approach is dependent on being able to simplify a complex model that one does not fully understand. In this paper, we argue that dealing with a formal, rather than a natural phenomenon, does not invalidate the normal scientific method and we show that this can be an effective approach to model simplification. In other words, to treat the complex model as if it was some natural phenomenon and to proceed to model it in the usual ways. Using this methodology, one can gain understanding of the complex model, and hence indirectly of the original natural phenomena. Furthermore, this allows additional validation of the hypothesised mechanisms, since each model can be used to check the other: hypotheses about the complex model’s processes and behaviour can be explored using the simpler model, and the robustness of the simpler model probed by doing experiments (on the complex model) as to the safety of the simplifying assumptions.

What do we mean by a “normal scientific method”? There has been a significant amount of philosophical discussion about this, from those that think that an identifiable normative standard should be imposed [2], to those that think that *any* constraint upon method is counter-productive [3]. Here we mean something much more mundane. Put simply, we mean some combination of the following strategies:

1. observation of the target phenomena to understand its mechanisms.
2. extraction of data from the target phenomena by measurement.
3. constructing models of the target phenomena.
4. assessing models by comparing their outputs with the data.

Each of these strategies can be equally applied to natural and formal phenomena. For example, if modelling the movement of ants one would take into account knowledge gained from observing them—e.g. that they are social animals and might follow each other. Similarly, if modelling a simulation one would naturally inspect the code and use one’s understanding of its mechanisms in a simpler model. Extracting data via measurement is much easier from a simulation than from natural phenomena since this can be done automatically using additional program code. Similarly comparing models using their output data is straightforward.

We do not presume to specify the correct, or most effective sequence, of the above strategies. Nor do we argue here for any particular rules about how the models in strategy (3) are formulated—whether from scratch or whether by adapting existing models. However, we do contrast this kind of approach with a purely deductive one, where one might attempt to reproduce some target phenomena using formal deduction from the formal structure of the original model.

Despite simulation reduction being a relatively under-studied issue [4], there has been some work on this within the simulation community. Here the emphasis has been mostly on complexity introduced as a by-product of simulation design and construction. For example, Innis and Rexstad [5] list 17 categories of simplification techniques, but most of these are either (sensible) engineering steps to prevent the introduction of *unnecessary* complexity or seek to exploit features characteristic of particular types of systems where simplifications are possible. However, they do include sensitivity analysis to see if some variables can be omitted and “Repro-meta modelling”—making a model of a model (as we do here).

Brook and Tobias [6] distinguish three kinds of model reduction: coding tricks; simplifications that preserve the output of interest exactly; and simplifications that preserve the output approximately. The first is of interest to anyone who is building a simulation—part of the range of techniques that are used to retain control over a developing complex simulation [7]. The second seeks an exact reduction. However, this tends to destroy the meaning of the content of the models they reduce (e.g. [8] approximates the input-output functions implicit in a model with a neural network and [9] simplifies by collecting model entities into abstract entities that enable a more efficient representation). It is the third category of approximating the output, that is of interest here.

There are fundamental limits to what automatic model reduction can achieve. Automatically checking whether one part of the code is functionally the same as another is, in general, undecidable (almost all general questions concerned with comparing the outcome of programs are undecidable, see any textbook on computability, e.g. [10] or read [11] for an examination of this specific question). Thus checking whether a simulation matches its specification is also undecidable. These sorts of results (which are simple corollaries of Turing’s undecidability theorem) mean that there will always be limitations to automatic techniques. This does not invalidate such methods but does imply that approaches that look for approximate and pragmatic simplifications will always be necessary. Machine learning techniques can automatically seek for representations of complex data and so could be applied to simulation outputs (final or intermediate) to infer models given their specific assumptions, but this does not result in simpler models from the point of view of a human trying to understand the dynamics [12]. The models may have a more uniform structure and less complex assumptions but the results are often so complicated as to be completely opaque [13].

Most of these techniques are not aimed at distributed phenomena but at simpler targets. The work of Ibrahim [14, 15] is an exception and addresses rule-based agent-based simulation. This proposes a framework for model reduction that (a) limits the reduction to answering particular ‘questions’ about the outcomes, (b) allows for approximate as well as exact methods, and (c) allows for a set of reduction strategies to be included. However this approach only partially works on more complex simulations.

We cannot prove that this ‘normal scientific method’ is a more effective way of model reduction than a deductive method. We can however, describe a case where this method was effective, that therefore supports the above approach of using a complex and a reduced simpler model in concert.

In this paper we will take as our example a complex model of voting, that considers the way various different social pressures lead to voter turnout. We first outline a complex model of voter turnout, which has been discussed elsewhere in more detail [16, 17]. Next we apply the ideas discussed above to this model and obtain a reduced model, the predictions of which are then compared to the original model. Finally we conclude with a general discussion of the potential of this approach and of further work on the reduced voter model. The S1 Text contains more details about the models and comparisons.

## Modelling voter turnout

In this section we will outline a complex model of voter turnout which has been constructed by a group of social scientists, in collaboration with one of the authors of this paper, to encapsulate the processes that are suggested by the literature on voter turnout [16, 17]. This is sufficiently complicated that the reduction process used to create a simpler model can be appreciated. Most modelling research on voter turnout, carried out by social scientists, is based on statistical analyses; there is no tradition in constructing models of voter turnout based on the interactions of many agents. There are, nevertheless, a number of studies available that model voting behaviour as a social influence process [18–24]. These tend to consider quite simple models that intend to capture, in a stylised manner, some aspect of the voting process or to reproduce some observed regularity.

**Fig 2:**
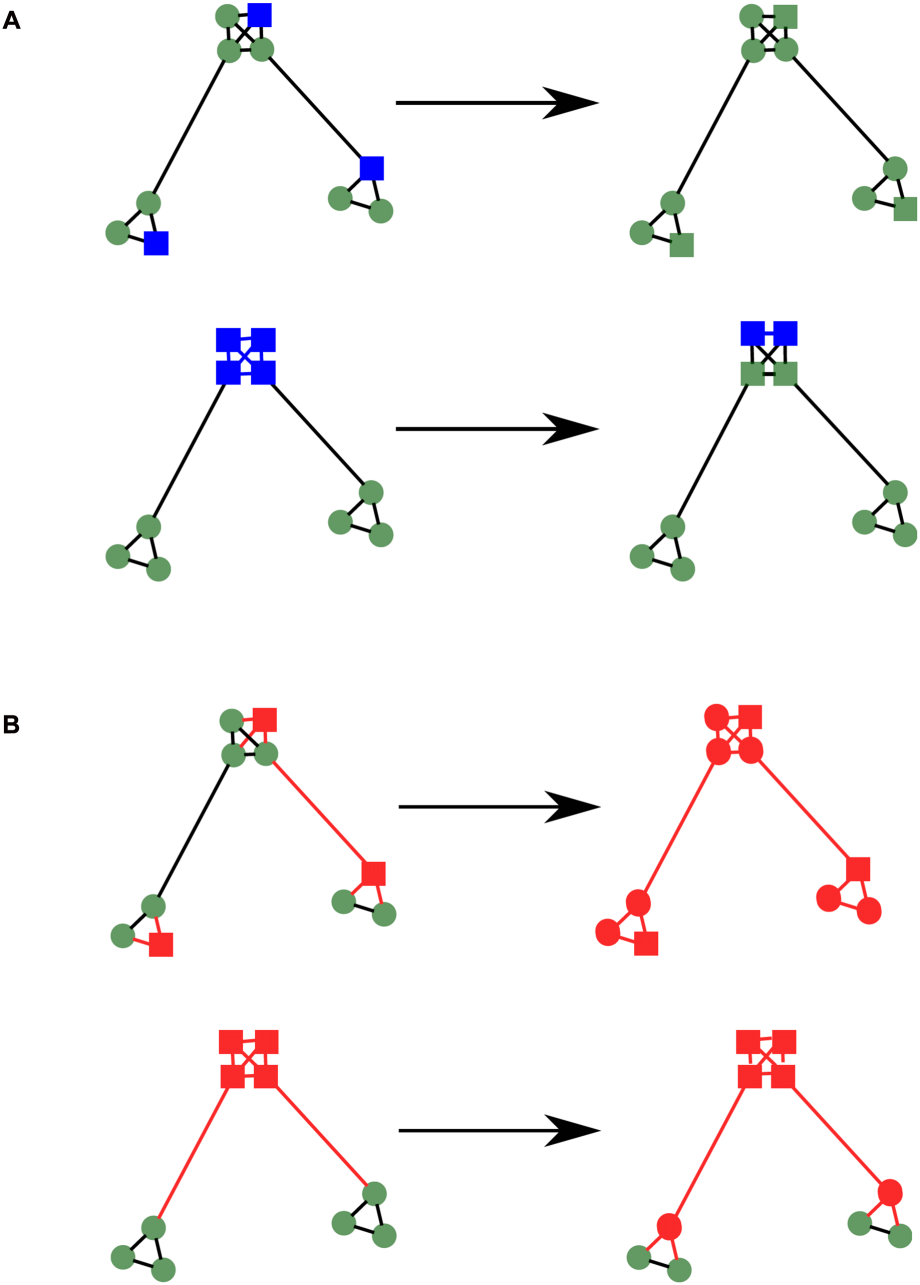
Diagrammatic representation of the full model processes. The main pathway is shown in blue, with additional factors in red, and development of the agent population in green.

In contrast, we start from a complex model of voting. This model was specified by social scientists to reflect the current micro-level evidence concerning how and why people vote. Below, we give an overview of its main mechanisms. The full model can be thought of as a core structure whereby a changing population of agents generate a social network, spread influence over this network, and finally decide whether or not to vote in a general election (see Fig 2). In addition to these basic structures, there are other sub-processes and feedbacks within these mechanisms.

### Population development and demographics

A population of agents occupy sites on a square lattice, corresponding to households, places of work or activities, schools or are simply empty. Agents have numerous characteristics (age, education-level, ethnicity *etc*.), and some of these may change during the simulation. Agents are born, age and die, immigrate and emigrate. Agents that are not born in the simulation (*i.e*. those initialised at the start of the simulation, or who are immigrants into the simulation), are created using demographic and socio-political data taken from the 1992 wave of the British Household Panel Study (BHPS) [25].

### Network formation and change

Agents create links, giving rise to several parallel social networks, corresponding to different types of relationships: partners, family, neighbours, via children at school, work colleagues and via mutual membership of activities. Partnerships and other friendships are formed primarily between agents that are ‘similar’ in terms of age, ethnicity, class and political identification. A ‘friend of a friend’ process occurs within each kind of network. Links can be also dropped. Agents can move house (for example when partnering) and can find and change jobs and activities.

### Influence spread

Agents initiate (political) conversations over these social networks (with different probabilities for different networks) with a frequency that depends on their level of interest in politics. This political interest level is determined by the number of instances of such conversations remembered. The conversations are forgotten over time with some probability. The recipient of each conversation is chosen at random from the agent’s links on the corresponding network. If the agent has civic duty then the recipient may gain civic duty and may also be persuaded to change their political identification. The number of conversations children have while living with their parents strongly influences their future political interest.

### Decision whether to vote

Agents intend to vote, for the party they identify with, if they have civic duty, or are in the habit of voting. They may also have an intention to vote based on whether their actions in previous elections have led to a desired outcome. Agents who intend to vote may be prevented from voting by confounding factors such as illness or newborn children. Agents will acquire voting habit if they vote in three consecutive elections and will lose it if they fail to vote in two consecutive elections.

We do not have space here for a complete description of this simulation (see S1 Text for more details). This is the point—this simulation is too complicated to completely understand, being formed of a complex mix of social processes that affect each other. Rather in this paper, we aim to describe how we sought to understand this by modelling it with simpler models, in a manner very similar to that if the target of analysis had been some natural phenomena. If our target for modelling had been some natural phenomena we would only had been able to give a similarly brief sketch of relevant aspects.

## Model reduction and comparison

To understand the complex model we constructed a series of reduced models, and then compared them to the original and each other. The procedure consisted of: creating reduced models (strategy 3, in the above) by removing or approximating aspects of the model that we expect to be less important; comparing the output with that of the original model (strategies 2 and 4); formulating hypotheses as to the origins of discrepancies between the models, based on careful observation and analysis of the mechanisms involved (strategy 1); iterating this procedure with new models.

After several iterations of of the above processes we found a reduced model that gave a sufficiently good fit in terms of outputs as compared with the original. The aspects of the original model which were removed or approximated are:

**Social network**: As a first approximation, we initially removed the social network, so that each individual may talk to any other agent in the simulation.
**Political parties**: Since we focus on turnout, we are not directly interested in which party was voted for. In the full model this does have an indirect effect on turnout, since agents may choose not to vote due to “rational considerations”, dependent on their judgement about the efficacy of their past history of voting on the desired outcome of the election. However, we did not expect this to play a large role in whether agents vote and hence removed parties and such “rational considerations” by agents.
**Children**: Since only adults vote, we eliminated the explicit raising of children. Instead we approximated this part of the model by creating all new agents as 18-year-old adults, with characteristics assigned to them based on approximations of the full model.
**Confounding factors**: In the full model agents that intend to vote may not vote due to a number of confounding factors (such as recent unemployment, illness, young children, etc.). We replaced these with a general probability of not voting, which is a function only of age.
**Population size**: We used a fixed population size, so that every emigration event is matched by an immigration event, and every death by a birth.

### Comparison between the reduced model and the full model (M_1_ with M_2_)

Although we compared the reduced and full model using several measurements, we will focus here on the dynamics of turnout, i.e. the proportion of the population who vote, since this was the principal focus when building the full model. In particular, we examine the range of voter turnouts for different values of the parameter ‘influence rate’, which scales the average number of times per year that agents initiate conversations.

**Fig 3:**
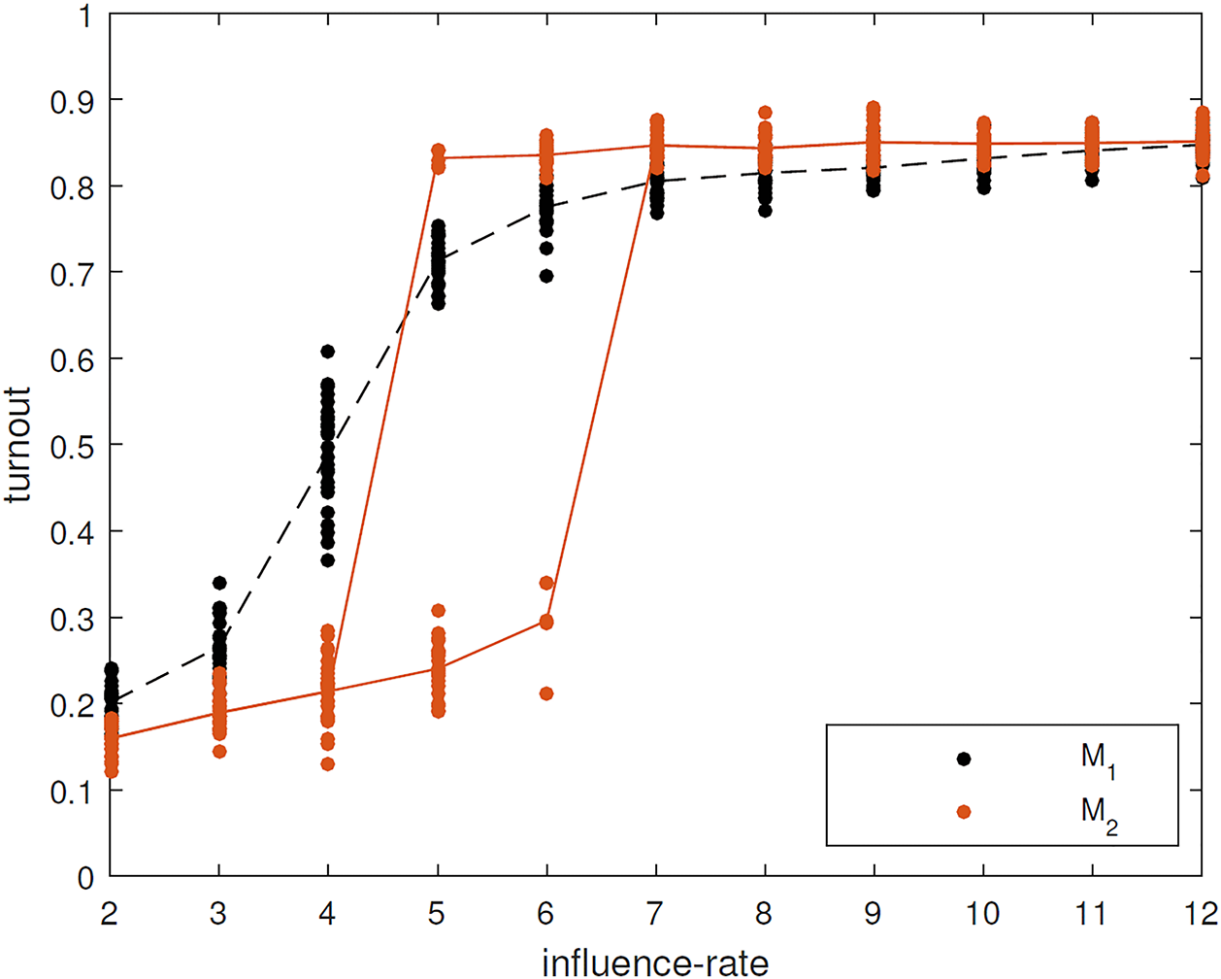
Comparison between the full model (black) and the reduced model (red). Ten different values of the influence rate parameter (from 2 to 11) are shown. For each one, the steady state value of the turnout obtained is shown for 25 realisations (dots), together with the mean values (lines).

We first compare the reduced model (denoted M_2_) described above with the full model (M_1_). Since we have removed many mechanisms we do not expect to obtain full agreement between the two models but since we retain the most vital parts of the full model we do expect to see some qualitative agreement. This comparison is shown in Fig 3.

Both models have two main ‘modes’: a high-turnout mode, corresponding to a high average number of conversations; and a low-turnout mode, corresponding to a lower number of conversations. The existence of different modes was only discovered through considering the reduced model, as it is over 1000 times faster to run. The two modes are due to the following: civic duty (acquired by talking to other agents that have civic duty) is highly correlated with voting, so that an agent with civic duty is very likely to vote; in parallel to this when agents are spoken to this increases their interest in politics, which then increases their likelihood of speaking to other agents. Thus increasing the influence rate parameter increases the overall amount of conversations in the model, both directly, through the effect of the parameter value, and indirectly, since agents that receive conversations are also more likely to initiate conversations. This feedback loop (Fig 2) amplifies the effect of the parameter and hence ‘locks-in’ a high level of turnout. Conversely, low civic duty levels and likelihood of conversations have the opposite effect.

We also find some quantitative agreement: in the high-turnout mode both models predict that roughly 80% of the electorate vote; while in the low-turnout mode voting is at around 20% (although the full model gives a somewhat higher level in the low-turnout regime). The parameter values in the reduced model are determined directly from the original model, and are not the result of ‘fitting’ the output. In the reduced model we find that for some intermediate values of the influence rate, the same initial conditions and parameter values can lead to either a high-turnout or a low-turnout mode in different simulations (bistability). In contrast, in the full model there is no region of bistability, instead intermediate influence rates lead to intermediate levels of voting.

The existence of high- and low-turnout regimes may be of practical interest. It suggests that the effect of efforts to increase voting might strongly depend on the parameter regime of the system. If we are close to the transition from low-turnout to high-turnout, then a small increase in the number of conversations people have about politics could be amplified to give a large effect on turnout, disproportionate to the initial effort of increasing the number of conversations. Conversely, if we are far from the region of transition, either deep in the low-turnout regime or in the high-turnout regime, then efforts to increase voting by increasing how much people speak about politics may have little effect.

From this point on, we add additional mechanisms to the reduced model and compare the outcomes to determine the effects and importance of these mechanisms. Each new mechanism will be described in the following sections and in the S1 Text, and will be given an acronym to distinguish the different versions of the reduced model. For example, the reduced model with the ‘clumped network’ (described below) will be referred to as M_2_+CN.

### Adding a synthetic “clumped” social network removes the bistability (M_2_ vs. M_2_+CN)

In the full model there are three networks generated by the model, each one used to carry out political conversations with a different probability. The most used network describes partnerships between couples, who live together on a single lattice site, perhaps with children or other adults. The second most used network depicts family relationships, which are typically between individuals that live on the same lattice site. Usually, each family is completely internally connected. Finally, there are long-range links, used less frequently, which give all other sorts of friendships (*e.g*. neighbours, work colleagues, school-friends *etc*.). Each of these networks are dynamic, evolving over time as agents age and change. The development of the networks includes mechanisms that take into account the homophily of the agents, so that agents are more likely to make friends with those who are most similar in terms of class, ethnicity, education level and political views. In contrast, in the reduced model agents talk with a randomly selected other agent in the model. This is equivalent to having a fully connected social network

**Fig 4:**
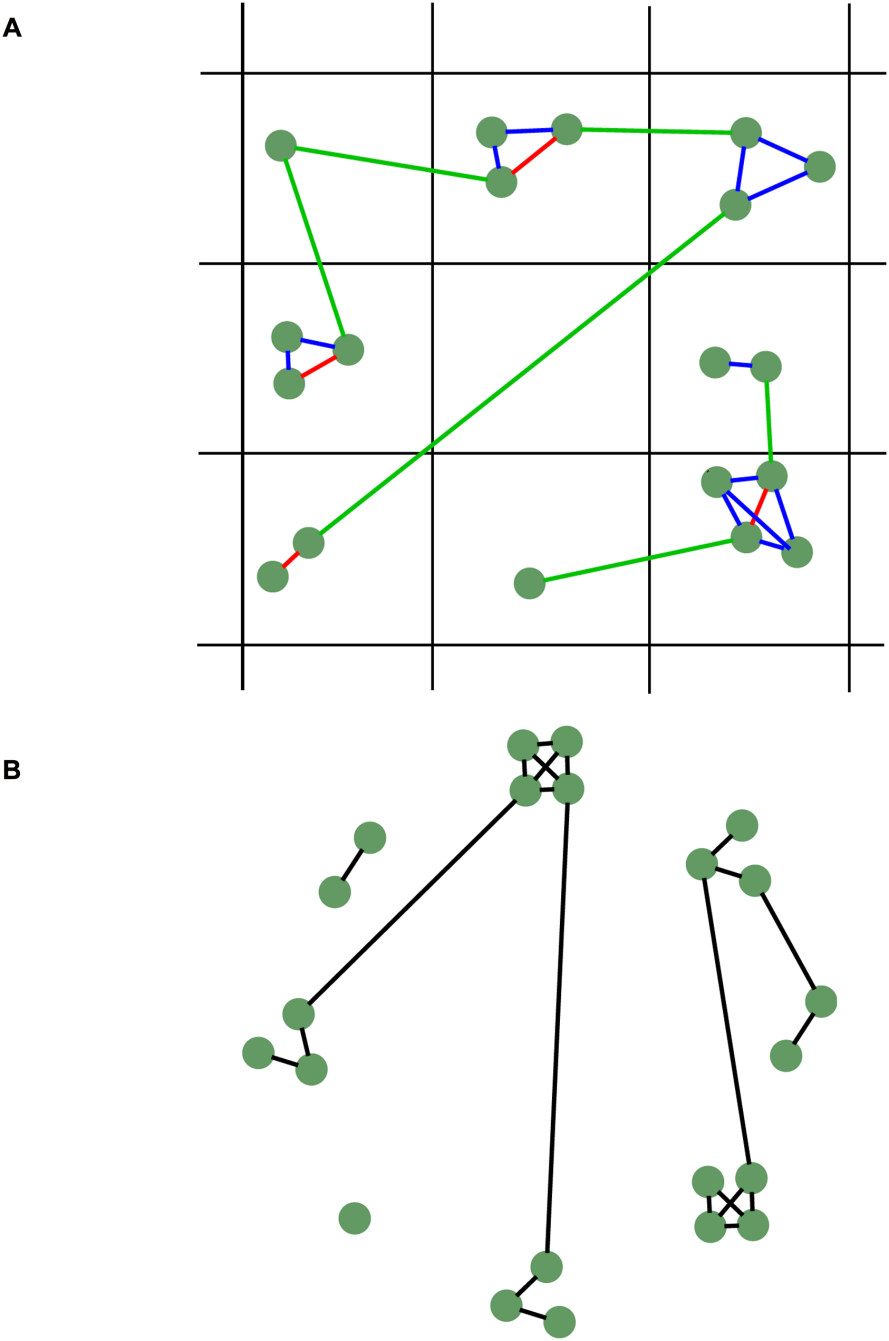
Schematic comparison between the full model network (left) and the synthetic network (network CN, right). Agents are displayed as green circles. Lines connecting agents represent social links. In the full model red lines represent partners, blue lines represent families and green lines represent other kinds of relationships.

We hypothesise that the important features of the network generated by the full model, with regards to turnout dynamics, relate to the general structure of the network, and not to specific characteristics of individual agents. To test this hypothesis we make a synthetic ‘clumped’ network (network CN) by creating totally connected groups of agents (representing households) and then rewiring some of these internal connections to create a few long-range links between groups, as illustrated in Fig 4. Thus there are two parameters for creating network CN: the average degree of an individual, and the probability of rewiring each link. A higher average degree creates a network that is more similar to that used by the reduced model, where agents may talk with any other agent. A higher rewiring probability makes the network more similar to a randomly connected network. It reduces the clustering [26] of the network, and represents the situation where households are less important compared to friendships outside the family group. The size of the initially fully connected groups was taken to be a uniform (discrete) random variable between 1 and 8, and each link was rewired with a probability of 0.12. In this way the degree distribution, the clustering coefficient and the proportion of local to long-range links were similar to those obtained in the full model (a precise fitting of these quantities is problematic since we are comparing a multi-level network to a simple one). This network CN is closely related to the so-called “caveman” graph [26]—it is a network that consists of “clumps” of well connected nodes with some long-range links between them.

**Fig 5:**
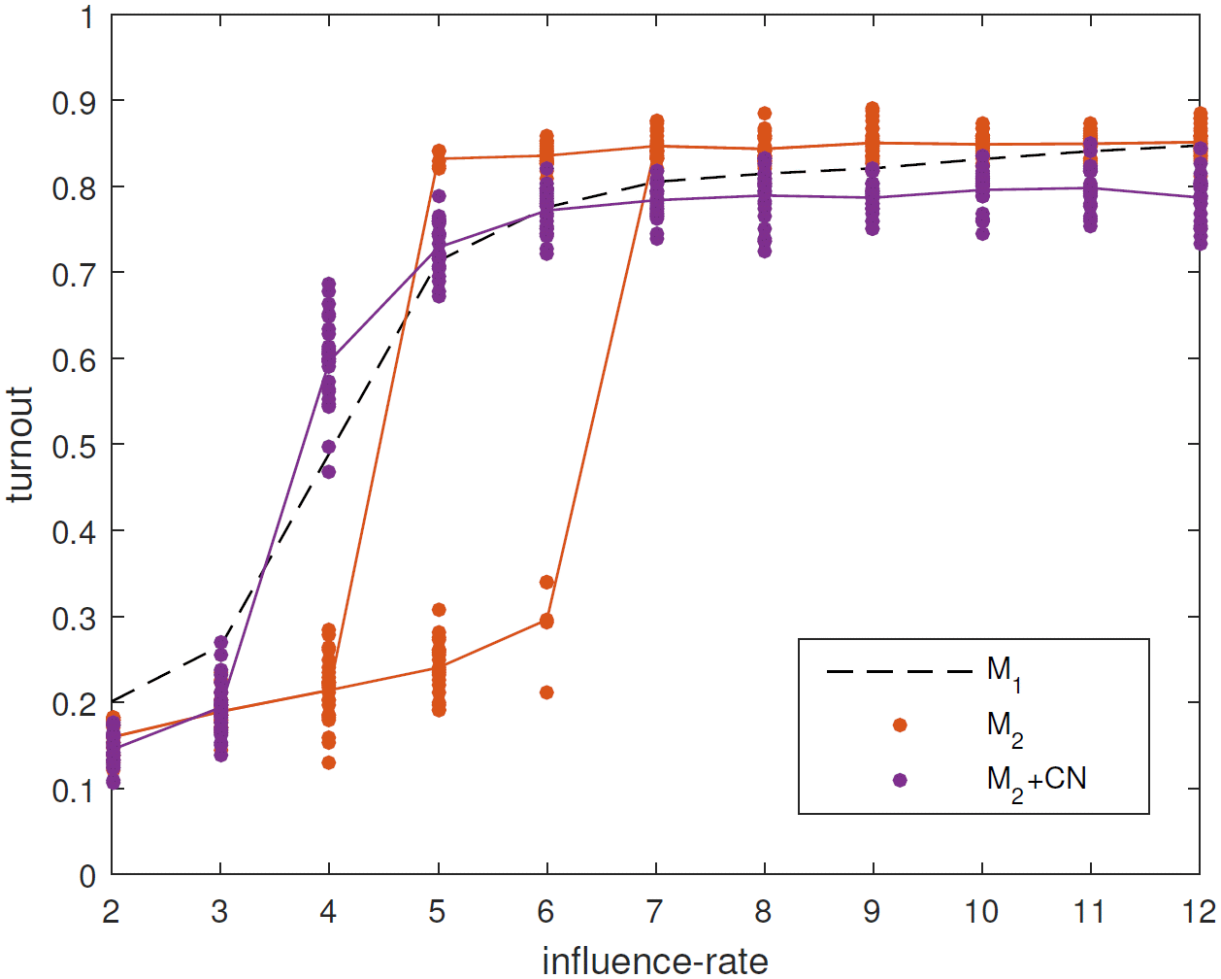
Comparison between the full model M_1_, (dashed black), the reduced model, M_2_ (red) and version M_2_+CN (purple). The synthetic network (network CN, see the main text) decreases turnout in the high-turnout regime and leads to a transition between low- and high-turnout at a lower influence rate.

The reduced model but with this network (M_2_+CN) gives a turnout similar to that of the full model. In particular, the turnout is slightly lower than without the network, and the transition from low- to high-turnout occurs at a similar value of the influence rate to the full model (Fig 5). Importantly, the bistability obtained with the fully connected network disappears. This network effect is due to the sparseness and the ‘locality’ of the network, and is further explored in S1 Text.

### Making the network dynamic leads to higher voting (M_2_+CN vs M_2_+CN+D)

**Fig 6:**
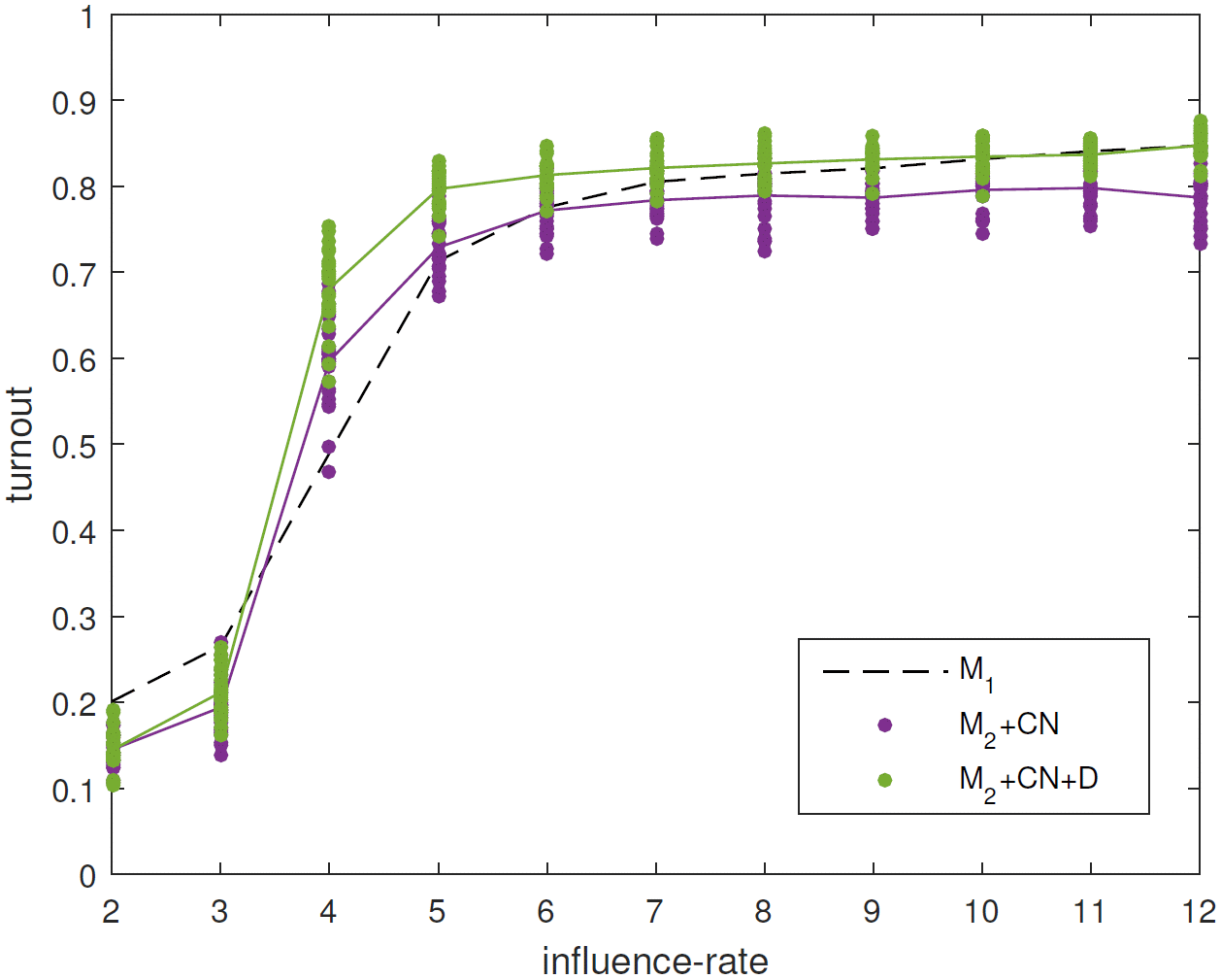
Comparison between the full model, version M_2_+CN (purple) and version M_2_+CN+D (green). Rewiring the network (with a probability of 0.15 per long-range link per year) increases turnout.

Using a synthetic social network (network CN) replicates the more gradual transition between low- and high-turnout regimes found in the full model, removing the bistability seen in the reduced model. However the turnout seen in the high-turnout regime is still lower than that found in the full model. One potential reason for this discrepancy is that the reduced model has a static network. To test this, we include dynamic network rewiring into the model from the previous section (M_2_+CN+D) in the following way. Every year, each long-range link is broken with probability 0.15 and one end of this link is reconnected to another agent selected at random. When we compare this to the model without dynamic rewiring (M_2_+CN), we find that the turnout is indeed increased in the high-turnout regime (Fig 6). In the low-turnout regime using a dynamic network does not significantly affect the turnout, since the probability that a low-interest agent is rewired to be connected to a high-interest agent is very low anyway. The increase in turnout due to dynamical rewiring was larger when the underlying network was sparse (network CN has an average degree of 3.5). The effect of dynamic rewiring is related to that found in [27], where rewiring facilitated the achievement of consensus in an Axelrod model [28]. Despite the improvement in agreement with the full model, the increased turnout in the high-turnout regime sometimes over-predicts turnout (Fig 6), indicating that additional mechanisms should be considered.

### Immigration by household leads to lower turnout than immigration by individual (M_2_+CN vs M_2_+CN+HI)

The last aspect we will consider is the implementation of immigration. Immigration in the model is rather high, with around half of the adult agents having been introduced as immigrants (note that immigrants correspond to all the agents not born in the population, not just foreigners). For this reason, we can expect that the particular form of immigration that we use will affect the results. In the reduced model each agent may emigrate with a given probability, and is replaced with a new immigrant agent. In contrast, agents in the full model immigrate and emigrate as households, so that all new immigrant agents begin the simulation living with other immigrant agents. This has the biggest effect when immigrants are drawn from a population that is significantly different from the native population.

**Fig 7:**
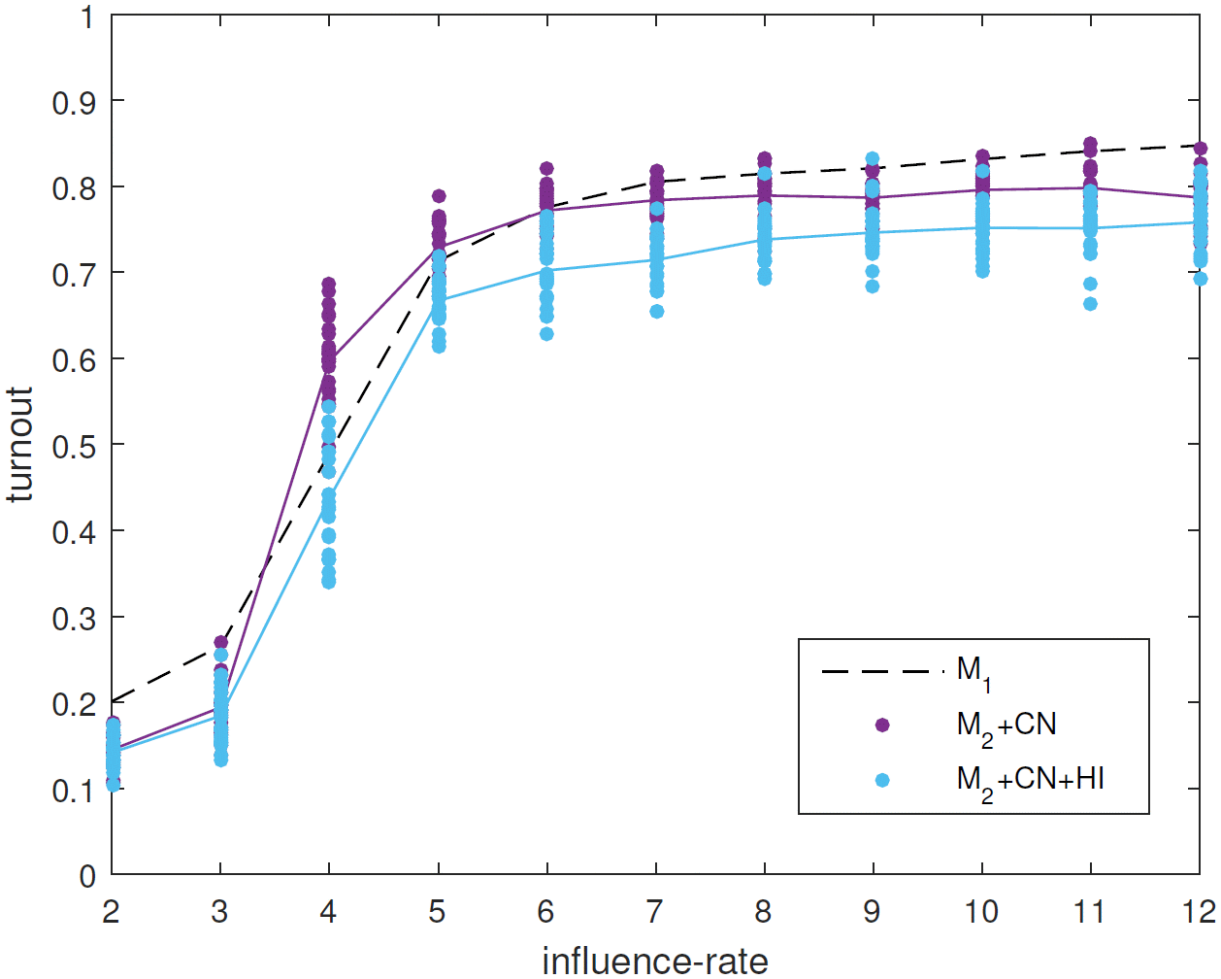
Comparison between version M_2_+CN (purple) and version M_2_+CN+HI (light blue). When immigration occurs by households turnout is reduced.

We compare the reduced model with the synthetic network (M_2_+CN) where immigration occurs individually to the same model but where immigration occurs by household (M_2_+CN+HI). This comparison is shown in Fig 7. We see that this does indeed lower the level of turnout. This can be explained as follows. In the high-turnout regime, where agents native to the model are very likely to have civic duty and high interest in politics, new immigrant agents are likely to have lower interest in politics and are less likely to have civic duty in comparison to the native population. If these new agents immigrate as a household, then the majority of their social links will be within that group, so that it is unlikely that an interested agent will initiate conversations with them. Thus agents forming a household of immigrants with low interest in politics are unlikely to increase their interest through conversations with native agents. Conversely, if agents immigrate individually and integrate into established groups of native agents, then these agents are likely to have higher interest in politics, and will initiate conversations with the immigrating agents, who will increase their own interest in politics. We therefore expect that including immigration by households in the high turnout regime will lead to moderately lower interest in politics and hence lower turnout overall due to lower turnout in the immigrant population. Thus using immigration by individual increases the effect that the higher-talking immigrants can have on the current native population. This mechanism is illustrated in the S1 Text.

Thus if one wishes to increase turnout in the general population, our model indicates that it is always better if immigrant agents are individually integrated into the general population. This result depends on the one-way nature of the influence included in the model, where agents can make others more interested in politics (and thus more likely to initiate conversations about politics), or teach others to have civic duty, but cannot make other agents less interested or less likely to have civic duty. While this is an assumption of the model, it is grounded on some evidence [29].

### Including both extensions results in a better fit with the full model (M_2_+CN+D+HI vs M_1_)

**Fig 8:**
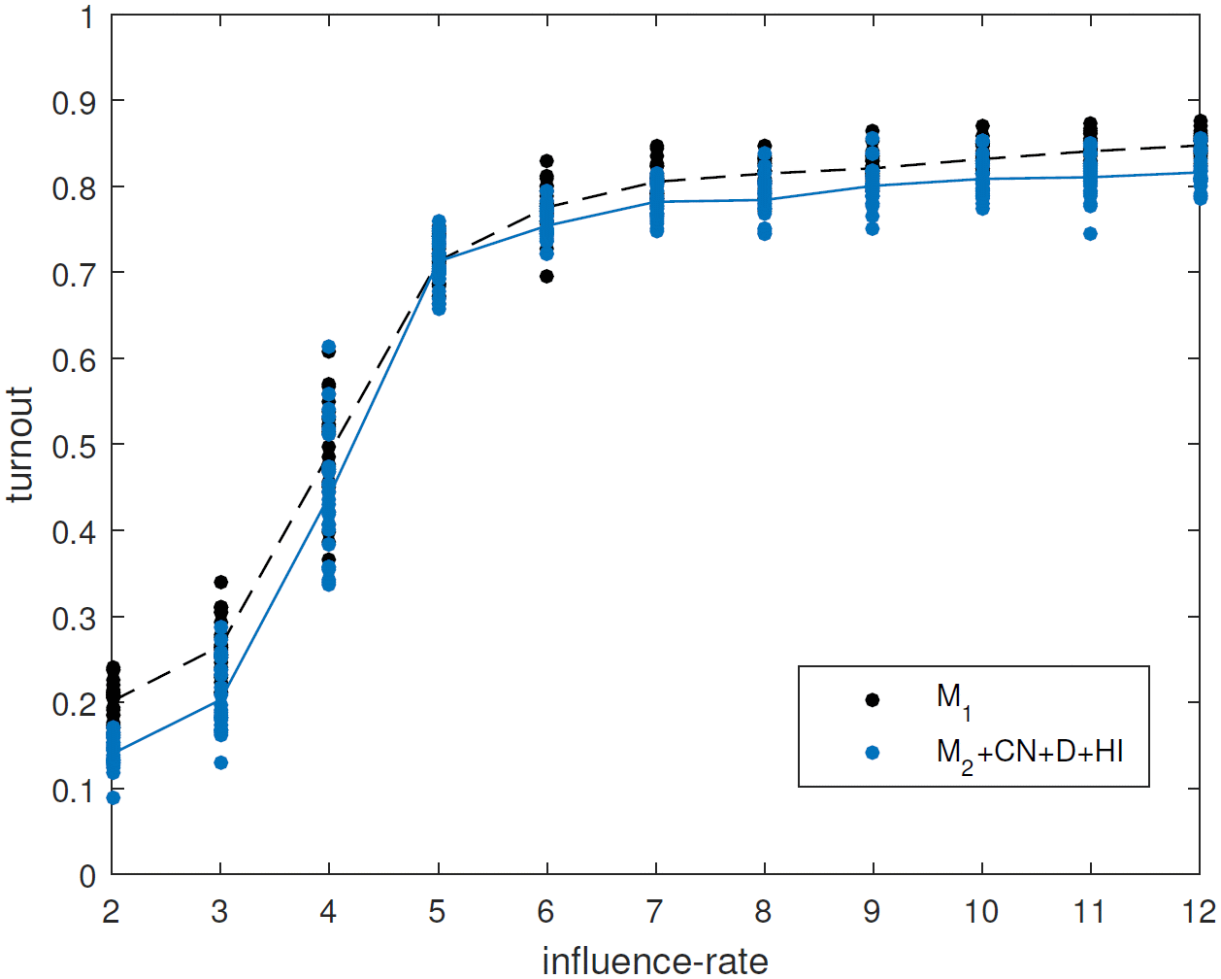
Comparison between original model (black) and version M_2_+CN+D+HI (blue). Ten different values of the influence rate parameter (from 2 to 12) are shown. For each one, the steady state value of the turnout obtained is shown for 25 realisations (dots), together with the mean values (lines).

In the preceding sections we discussed the impact of two mechanisms: allowing dynamic network rewiring during the simulation; and implementing immigration by households. Here we include both of these mechanisms simultaneously (M_2_+CN+D+HI) and demonstrate that with both of these mechanisms the model compares well with the full model at all values of the influence rate parameter (Fig 8). Thus we have substantially improved the fit of the reduced model for the particular target of turnout dynamics and for the parameter ranges considered. It might be that different reduced models will be suitable for different output targets and parameter ranges.

Although version M_2_+CN+D+HI includes more mechanisms than the fully reduced model, M_2_, it is still significantly simpler than the full model. Version M_2_+CN+D+HI runs approximately 1000 times faster than the full model (see S1 Text). Exploration and analysis of the reduced model is substantially easier and faster than with the full model, although we have maintained a relatively close correspondence between the two.

## Discussion

In this paper we have described a method for simplifying and understanding a complex model using a series of simpler models, and have used this method to better understand an intricate agent-based model of voting. We have thus demonstrated the effects of the different mechanisms included in the model, and have shown that significant simplifications can be made without compromising the target results over a particular range of parameter values. We believe that this approach can be applied to the analysis of other complex phenomena that cannot be adequately represented using a single simple model. The approach detailed here provides a structure to facilitate interdisciplinary collaborations between data-driven modelling, requiring a high level of detail, and an analytical approach, requiring simpler models that are more amenable to systematic analysis. In this way, insights obtained in the simpler models can be seen to be relevant to the more complicated models, and the systems they describe.

We stress that our model-reduction approach involves no substantial data fitting. Most of the parameter values of the reduced model are directly given by those of the full model. When there is not a direct correspondence of parameters, such as for parameters of the reduced network (which is a simple network as opposed to the multi-level character of the full model’s network), the parameter values are chosen so that the microscopic characteristics (such as degree distribution or proportion of long-range links) mimic those of the full model.

The kind of process described above has resulted in simpler models that are different than one might invent in a one-step modelling process—the composition of these models was guided by what was in the complex model and what turned out to be significant for the narrow question of turnout dynamics. The simpler models have resulted in insights into the workings of the complex model—insights that would have been difficult to obtain through direct simulations due to the slowness of execution of the complex model and its very large parameter space. Thus the importance of the detail of the social network has been revealed for the transition between high-turnout and low-turnout regimes, and the potential different impact of different modes of immigration highlighted. The process of developing all these models and comparing different variations is relatively time consuming, but one ends up with a chain of related models that combine some of the advantages of simplicity with the assurance of relevance. We have recently developed a voter model which is even simpler than the one presented here and which is amenable to a mathematical analysis. This was constructed as a further reduced version of *M*_2_ described in this paper. This then is the next link in the chain, and will be discussed in detail elsewhere.

## Supporting Information

S1 Text Further details about the model analysis and the reduced and full model specifications. (PDF)
