# Correction: Staged Models for Interdisciplinary Research

**DOI:** 10.1371/journal.pone.0162151

**Published:** 2016-08-25

**Authors:** The PLOS ONE Staff

The images for Figs 1 and 2 are incorrect and the legend for Fig 4 is incorrect. The corrected figures and captions are provided here.

**Fig 1:**
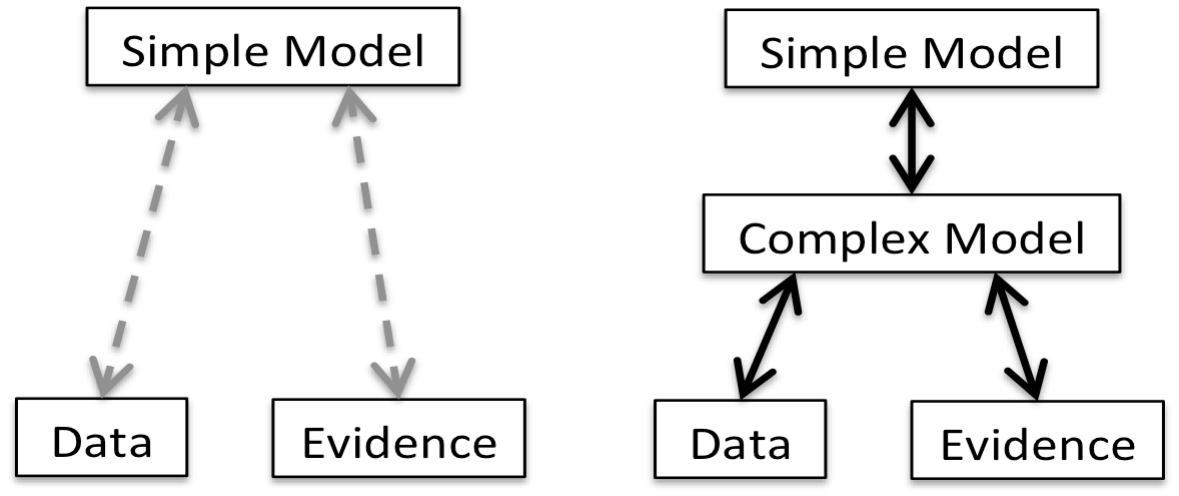
From a single to a multi-stage abstraction process.

**Fig 2:**
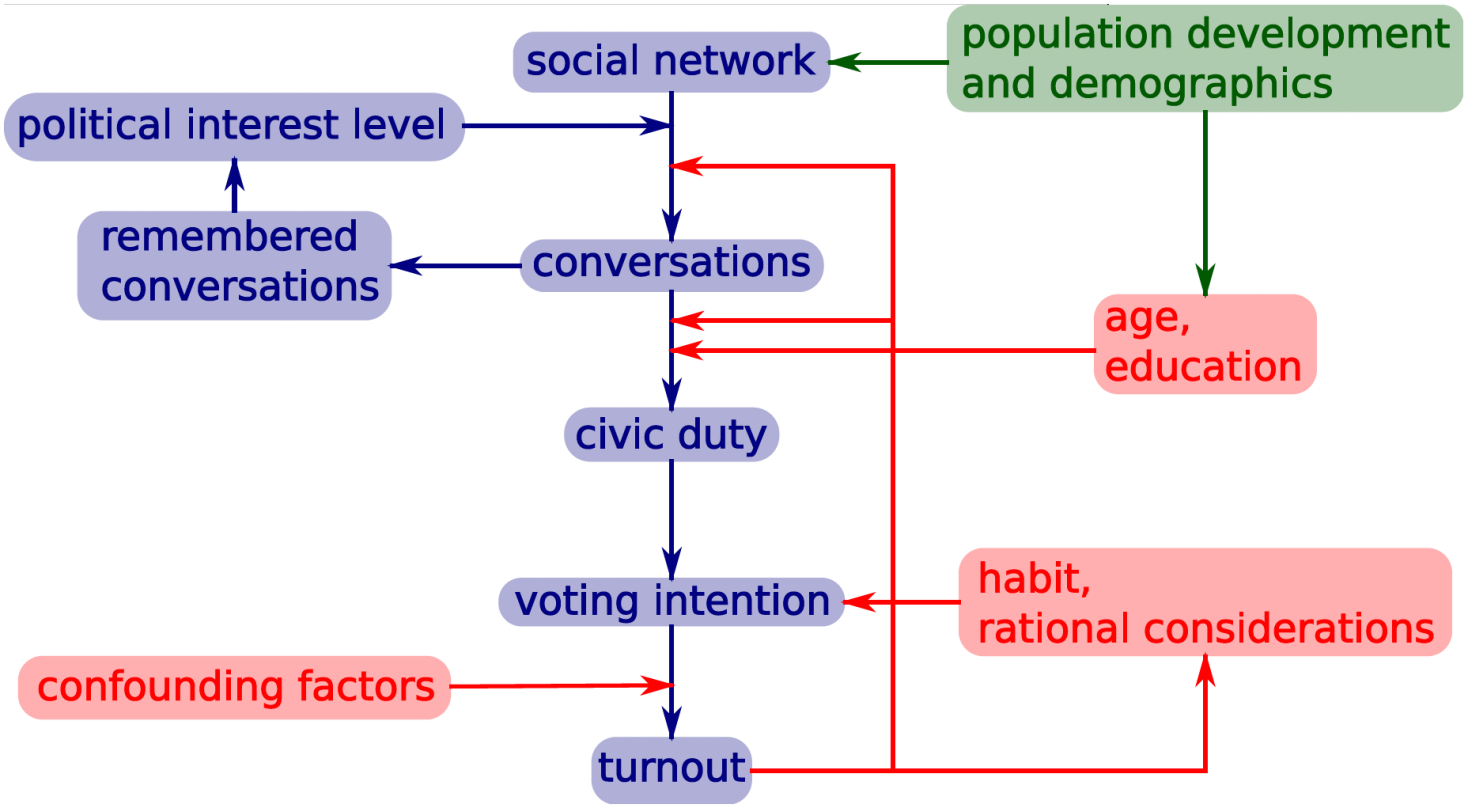
Diagrammatic representation of the full model processes. The main pathway is shown in blue, with additional factors in red, and development of the agent population in green.

**Fig 4:**
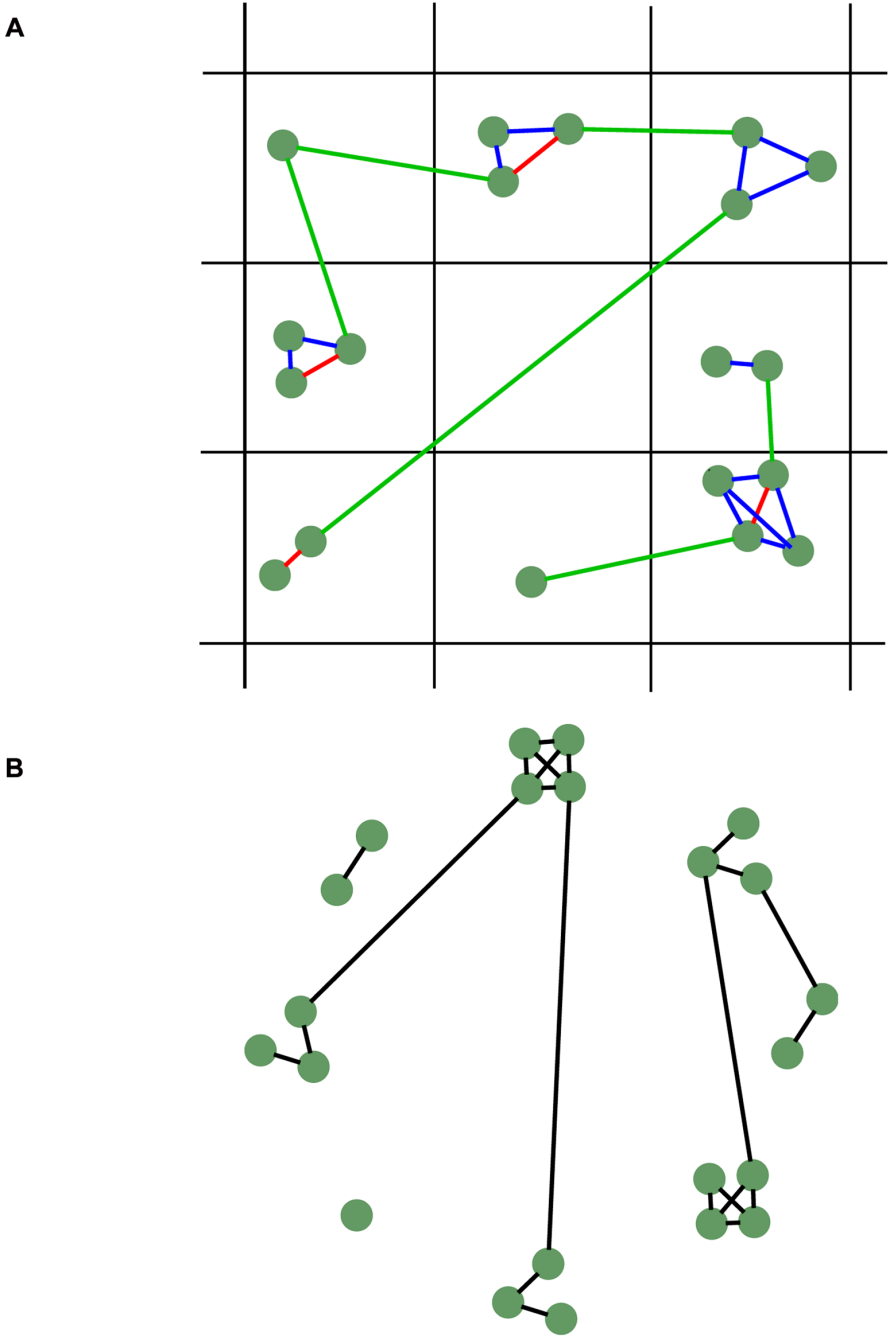
Schematic comparison between the full model network (A, above) and the synthetic network (network CN, B below). Agents are displayed as green circles. Lines connecting agents represent social links. In the full model red lines represent partners, blue lines represent families and green lines represent other kinds of relationships.
