# Correction: Correction: Staged Models for Interdisciplinary Research

**DOI:** 10.1371/journal.pone.0165250

**Published:** 2016-10-18

**Authors:** The PLOS ONE Staff

## Notice of Republication

This article was republished on September 19, 2016, to correct an error in the figures provided in the correction article. Figure 1 in the correction article was incorrect. The publisher apologizes for the errors. Please download this article again to view the correct version. The originally published, uncorrected article and the republished, corrected articles are provided here for reference.

## Supporting Information

S1 File Originally published, uncorrected article. (PDF)

S2 File Republished, corrected article. (PDF)
