# Supplementary material for: Staged Models for Interdisciplinary Research
Source: PLoS One. 2016 Jun 30;11(6):e0157261. doi: 10.1371/journal.pone.0157261 (PMC4928782; doi:10.1371/journal.pone.0157261)
Supplement: S1 Text — (PDF) [file pone.0157261.s001.pdf]

Supplementary Information: Staged Models for Interdisciplinary Research

Luis F. Lafuerza[1], Louise Dyson[1,a], Bruce Edmonds[2,*], Alan J. McKane[1].

1 Theoretical Physics, School of Physics and Astronomy, University of Manchester, Manchester M13 9PL, UK
2 Centre for Policy Modelling, Manchester Metropolitan University, Manchester, M15 6BH, UK

a Current address: Mathematics Institute, University of Warwick, Coventry CV4 7AL, UK
*** bruce.edmonds@gmail.com**

Further details on the model analysis

Effects of a static network

In the main text we show that, while a fully connected network leads to a sharp transition with a region of bistability, a (fixed) network made of small, strongly connected communities with a few links between them leads to a smoother transition in which the bistability region disappears (see Figure 5 of the main text). More precisely, the network considered was made of fully (internally) connected groups whose size was taken to be a uniform (discrete) random variable between 1 and 8; then, each link was rewired with a probability of 0.12. More generally, we can consider a family of networks of this type parametrised by the average size of the groups, s, and the rewiring probability, r. Here s controls the average degree (or, equivalently, the network density or connectivity), and r the degree of 'clumpiness' (related to the modularity and the clustering coefficient). Using this family of networks, we can explore the effects of the connectivity and the 'clumpiness' on the dynamics. We find that when s or r are increased, the dynamical results are more similar to those obtained for the fully connected network, and the bistability region is recovered, as illustrated in Figure 1. In this way, it is clear that the connectivity and the 'clumpiness' of the network are crucial characteristics in determining the behaviour of the model, particularly in the region of intermediate social influence. This prediction was confirmed in the full model, M_1, where it was found that artificially increasing the number of links could give rise to bistable behaviour.

Effects of the immigration implementation

In the main text we show that when immigrants enter the simulation via whole households the overall turnout decreases (compared to the case in which immigrants enter individually). The mechanism giving rise to this result is illustrated in Figure 2. The origin of this effect lies in the asymmetry of the social influence process, whereby highly interested individuals can increase the interest of less interested individuals but less interested individuals cannot decrease the interest of highly interested individuals. Under these circumstances, a higher level of overall interest is achieved when highly

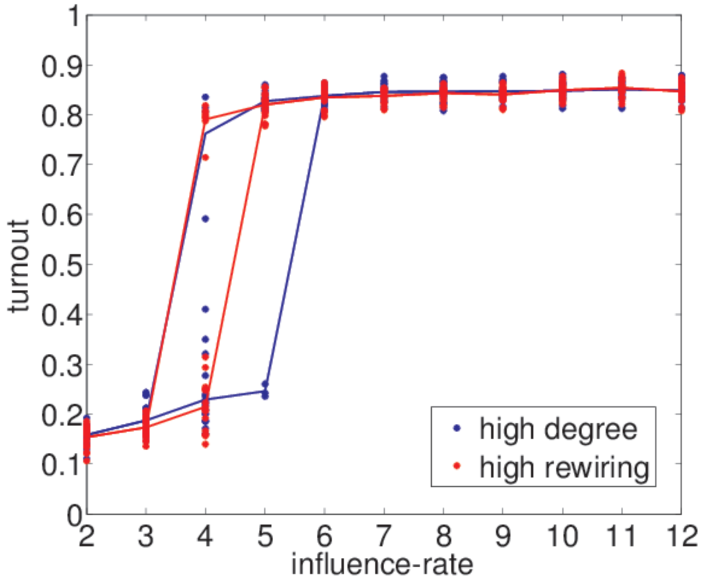

Figure 1. Using a network with either high degree (blue) or high rewiring probability (red) regains the bistability observed in the reduced model. Both simulations use model M_2. The high degree network has degree = 65 and rewiring probability = 0.15. The high rewiring network has degree = 15 and rewiring probability = 0.45.

interested individuals are more connected to less interested individuals, while a lower level of interest is achieved if highly interested individuals are more connected with other highly interested individuals. This is so because the influence of highly interested individuals is 'wasted' if they talk to one another, while it has a larger effect if it is concentrated on less interested individuals. When the immigrants are different to the incumbent population, it will boost turnout if they tend to be connected to the incumbent population; if immigrants have higher interest they will be able to pass this interest to the rest of the population; if immigrants have lower interest they will have the chance to increase it via contacts with the rest of the population.

Runtime comparison

To assess the difference in computational demands of the full and the reduced model, we compared the (real) time needed to run the models on a standard desktop computer. We used the parameter values employed in the main text, as indicated in Table 1, varying the parameter influence rate, which controls the overall number of conversations in the population. We compared the full model, M_1, with version $M_2 + CN + D + HI$ of the reduced model, which is the more complex of the reduced models. The reduced model was implemented in C programming language (while the full model is written in NetLogo). The results are shown in Figure 3.

Reduced model description

Each agent (with index $i = 1, \ldots, N$) has the following list of characteristics, some of which may change over time:

binary variables: civic duty ($CD(i)$), turnout (in last election, $v(i)$), habit

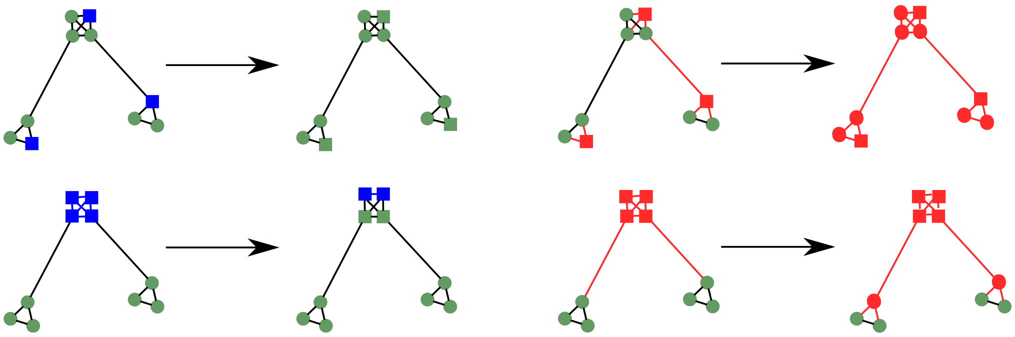

Figure 2. A schematic representation of the effect of immigration by individual vs. immigration by household: (left) when immigrants have a lower political interest and a lower chance of having civic duty than the general population and (right) when immigrants have a higher political interest and a higher chance of having civic duty than the general population. In each diagram, native agents are represented by circles and immigrants by squares. Political interest is shown in red (high interest), green (normal interest) and blue (low interest). In both cases immigration by individual leads to a higher interest in the population.

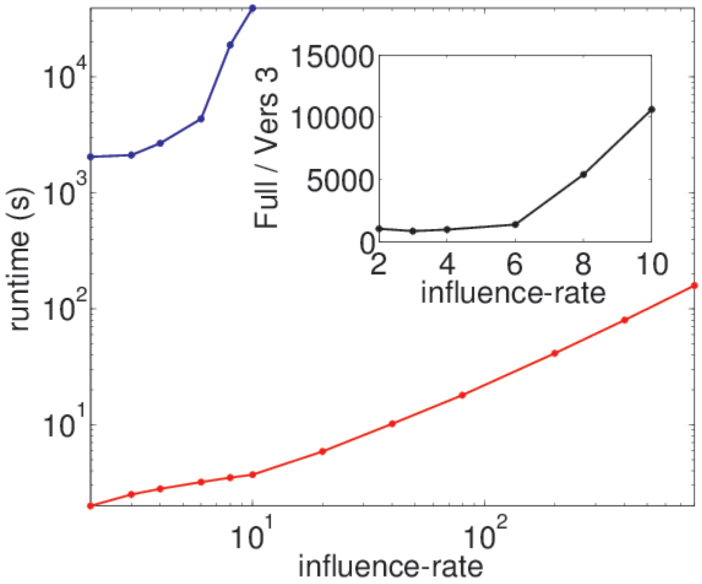

Figure 3. Run time comparison between the original model M_1 (blue) and the reduced version $M_2 + CN + D + HI$ (red). The time taken to run the simulation for different values of the influence-rate parameter for the original model, M_1, (blue) and version $M_2 + CN + D + HI$ of the reduced model (red). Note that for values of influence-rate above 10 simulations of the original model did not successfully complete. The inset shows the quotient of the two simulation times. Simulations were performed on a standard desktop computer.

$(h(i))$, post-18 education $(e(i))$;

integer variables: (political) interest level $(l(i))$, minimum interest level $(m(i))$, age (in years, $a(i)$), number of remembered (political) conversations $(c(i))$.

The main parameters of the model are:
Influence rate K, scales the number of (political) conversations per year.

Probabilities of initiating a conversation $\mathbf{p}_c(l, v)$.
Probabilities of gaining and loosing civic duty.
Thresholds on the number of conversations needed to increase the interest level Th_α.
Probability of forgetting a conversation $p_f(l)$.
Death probability $p_d(a)$.
Emigration probability p_e.
Probability of not voting due to confounding factors $p_c(a)$.

Initialisation procedures

Agents are initialised using data from the British Household Panel Study (BHPS) [1]. The same procedure initialises immigrants into the model, using the subset of the BHPS corresponding to survey responses from immigrants. This procedure sets the civic duty, turnout, habit, post-18 education, interest level and minimum interest level, with some of these characteristics being inferred using proxies for the required information. Agents initially have no remembered conversations, and an age drawn from a uniform distribution between 18 and 70 (to initialise the model) and between 18 and 48 (for later immigrants into the model). Agents born during the simulation are initialised at age 18 (we do not explicitly model their growth until age 18), and education with probability 0.3. Their interest level and minimum interest level is equal to their education, and they are assumed have no civic duty, habit or remembered conversations and not to have voted in the last election.

Main loop

The following processes happen in a loop until the required timepoint is reached. All rates are given in Table 1. **Each year:**

Each month:

Carrying out conversations: For each agent, this section is run $\lfloor K/12 \rfloor$ times plus one time extra with probability $K/12 - \lfloor K/12 \rfloor$.

The agent has the chance to initiate three conversations, with probabilities $\mathbf{p}_c(l(i), v(i))$ each with a random other agent.

Agents (with $l(i) > 0$) receiving a conversation (from an agent with civic duty), acquire civic duty with probability $p_{acd}(e(i), v(i))$.

Updating interest levels:

If $l(i) = 0$ and $c(i) > Th_0$ then set $l(i) = 1$ and $m(i) = 1$.

Else, if $c(i) > Th_h$ then set $l(i) = m(i) + 2$.

Else, if $c(i) > Th_l$ then set $l(i) = m(i) + 1$.

Updating civic duty: Agents lose civic duty with probability, $p_{lcd}(a(i), e(i))$, dependent on their age and education.

Forgetting conversations: Agents forget conversations that happened more than one year ago, with probability, $p_f(l(i))$, per conversation, dependent on the agent's interest level.

Birth/death: Each agent dies with a probability, $p_d(a(i))$, dependent on their age, and is replaced by a new agent by the 'birth' process (described in the Initialisation procedures).

Immigration/emigration: Each agent emigrates with a probability $p_e = 0.015$ and is replaced by a new agent by the 'immigration' process (described in the Initialisation procedures).

Ageing: Agents age by one year

Every 5 years there is an election:

Agents with civic duty or habit vote unless 'confounded' (due to illness or other factors) with probability $p_c(a(i))$, dependent on their age.

Agents gain habit if they vote in 3 consecutive elections.

Agents lose habit if they do not vote in 2 consecutive elections.

Here $\lfloor x \rfloor$ denotes the integer part of x, that is, the largest integer less than or equal to x.

Table 1. Parameter values of model M_2.

Parameter name	Value	Meaning
N	480	population size
$p_d(a)$	a function of age derived from mortality tables	death rate
p_e	0.015	emigration rate
K	$K \in [2, 12]$	influence rate
$p_c(l,v)$	$\mathbf{p}_c(2,0) = [0.0100, 0.0500, 0.1500]$<br>$\mathbf{p}_c(2,1) = [0.0600, 0.1000, 0.1800]$<br>$\mathbf{p}_c(3,0) = [0.0600, 0.1925, 0.3795]$<br>$\mathbf{p}_c(3,1) = [0.1540, 0.2800, 0.3900]$<br>$\mathbf{p}_c(4,0) = [0.2000, 0.4750, 0.5134]$<br>$\mathbf{p}_c(4,1) = [0.3232, 0.5680, 0.5370]$	probability of initiating a conversation ($\mathbf{p}_c(l,v) = [0,0,0]$ if $l \leq 1$)
$p_a cd(e,v)$	1-(1-0.25(1+e))(1+v))(1-0.125(1+e))(1+v))	probability of acquiring civic duty
Th_0	5	threshold for increasing interest level to 1
Th_l	2	lower threshold for increasing interest
Th_h	5	higher threshold for increasing interest
$p_{lcd}(a,e)$	$0.01/(12(1+e))$ if $a \geq 25$<br>0 if $a < 25$	probability (per month) of losing civic duty
$p_c(a)$	0.077 if $a \leq 75$<br>$0.077 + (1 - 0.077)0.9^{(a-75)(a-74)/2}$ else	probability of not voting due to being confounded
$p_f(l)$	0.2 if $l = 0$<br>0.5 if $l \geq 1$	probability (per year) of forgetting a conversation

Description of the Full Model

Here we give more detail about the full model (M_1). This description will follow the "ODD" protocol for this [2]. The full code, a complete description of the details of the model and a sensitivity analysis can be found at [3].

Overview

Purpose of Model

This is intended as a consistent, detailed and dynamic description, in the form of an agent-based simulation, of the available evidence concerning the question of why people bother to vote. This integrates a variety of kinds and qualities of evidence, from source data and statistics to more qualitative evidence in the form of interviews. The model is being developed following a KIDS rather than a KISS methodology, that is, it aims to be more guided by the available evidence rather than simplicity [4].

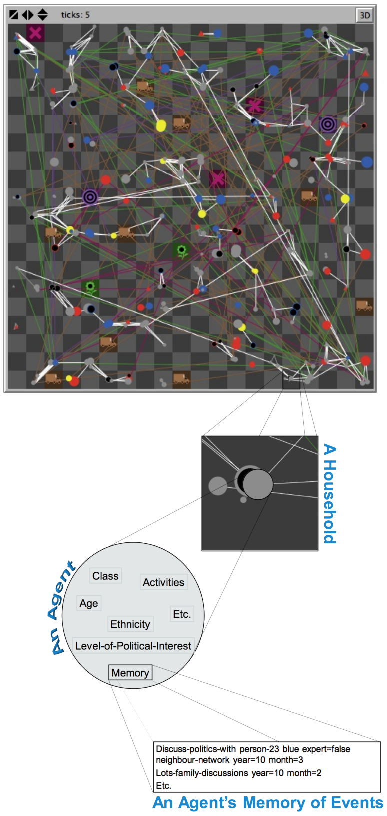

Figure 4. An illustration of the model elements. Each patch represents a household or other location (place of work, school, or kind of activity). The circles or triangles on patches are the agents of a household. The links between them social networks of different types. Agents have a number of attributes, including: their ethnicity (shape), their political leaning (colour), and their age (size). Other agent attributes include class, level of political interest, and which activities they belong to. They also have a (partial) memory of past events, including who they voted for and the political discussions they have had.

Entities, state variables, scales

The model is based around a 2D grid of locations, each of which may be a: household, place of work, school, activity (two kinds) or empty. Households consist of a number of agents which each represent a single person. Agents are born, age, partner, have children and die as the simulation progresses. Agents have a large number of characteristics, but these include: a memory of past events, a party affiliation (or none), a set of family relationships (children, partner, and/or parents) and social connections with other agents. It is over the network of social relationships that influence occurs in the form of events that represent communication about political or civic matters. The agents are influenced over time via these communications. When an election occurs, these influences, along with other factors, affect whether an agent votes and, if so, for which party. The collected votes are endogenously summed to give the election result, which might affect whether agents consider voting again. These elements are illustrated in Figure 4.

Places of work, schools and activities are place-holders. They do not change or move

(unlike the households). Their only characteristic is their membership (who works there, which children go to school there, which are members of an activity). A household is simply a container for the agents who form that household.

Agents are the primary elements in the simulation and have many characteristics, including:

- Age: the number of simulation years since they were born
- Ethnicity: their ethnicity (majority, invisible minority, or visible majority)
- Partner: the agent who is their partner (if any)
- Children: their children (if any)
- Older-relations: adults in their household when born (if any)
- Parents: their parents (if any)
- Employed?: whether they are employed
- Last-lost-job: when they lost their last job (if unemployed)
- Voted?: whether they voted in the last election
- Voted-for: who they voted for in the last election (if any)
- Year-last-child: when they had their last child (if any)
- Immigrant-gen: a number indicating if they are direct immigrants (0), the next generation (1), etc.
- Class: Which of the 5 classes the agent belongs
- Moved-out?: whether the agent has left their birth home (ever)
- Ill?: whether the agent is currently ill
- Post-18-edu?: whether the agent has acquired a post-18 education
- Civic-duty?: whether the agent has a sense of civic duty (with respect to voting)
- Interest-level: the level of interest an agent has in politics, one of: not noticing politics, noticing politics, taking a view on issues, politically interested, and politically involved
- Party-habit?: coming from voting for a party in consecutive elections
- Gen-habit?: whether the agent has the habit of voting
- The number of consecutive elections in which the agent has voted
- The number of consecutive elections in which the agent has voted for the same party
- The number of consecutive elections in which the agent has not voted
- Memberships: those schools, places of work or activities that an agent belongs to
- Social links: those other agents with whom they might (if both were inclined) have a political discussion

- Memory of events: such as recent political conversations they have had, whether they felt satisfied with voting, or whether there was lots of political discussion at home Other characteristics exist for the purpose of simplifying programming, debugging and collecting statistics and these are not listed. Note that currently agents are not sexed so partnerships can be formed between any two agents.

Process overview, scheduling

The simulation is initialised at the start. Then the simulation proceeds in discrete time steps, one step usually representing each month in a year. Each time step the following stages are carried out.

1. External immigration and households moving into area from outside of the UK sampled from immigrants in BHPS sample, unless grid is full
2. Internal immigration and households moving into area from inside of the UK sampled from all BHPS sample, unless grid is full (remixed in terms of given majority/minority mix)
3. Emigration and households moving out of the area
4. Birth and death probabilistically using statistics
5. Forgetting and things being lost from the endorsements of agents at different rates, e.g. remembrance of conversations
6. Network-changes and social links to other agents and activities made and broken
7. Partnerships are formed, move to live together if possible
8. Partnerships dissolved, one partner moves out
9. Household might move within the simulation area
10. Have conversations and hold conversations over the social network, influencing others in the process, the frequency of this is adjusted using the influence-rate parameter
11. Updating agents' attributes in terms of: noticing politics, interest level, and civic duty
12. Once a year update: the party preference, party habit and generalised habit
13. Drift-process and shift of voters into and from each political party by a drift process: voters for ruling party (not very interested in politics) drift away to grey, some grey drift to a party
14. (During the short run-up to an election): politically involved agents probibilistically contact random adult agents and have a political conversation with them which may increase their probability to vote.
15. During an election tick:
    - Determine an agent's intention to vote based upon: their satisfaction with their past experience of voting, whether they have a sense of civic duty, whether they have acquired the habit of voting, whether they feel an identification with a particular party, their level of interest in politics

- Factor in some of the effects of confounding factors (such as having recently moved, or having a child under 1) and record some statistics about consecutive voting etc.
- If it is to occur (determined by the simulation settings) the effect of party mobilisation efforts are computed
- For all those going to vote, then may drag others to vote with them
- The voting process itself happens including recording information of who voted and why (for the model analysis)
- The election result is determined via endogenous voting by agents (although it could be exogenously fixed if this is set)

16. Updating various plots and statistics for output about what is happening in the simulation

For each of these stages agents are fired in a random order (newly random each time and process). In most of these processes the update for each agent has no immediate effect on any other agent, so these agent processes are effectively in parallel. Similarly most of these stages could be done in any order with very little impact on the outcome, the exception being the sub-stages of voting (item 14 above).

Design

Basic principles

The starting point for the model design was a collection of 54 “causal stories” about behaviour that might be relevant to whether people bother to go and vote. Each such story traces a single causal thread through the complexity of social and cognitive processes whilst letting the context of these be implicit and whilst ignoring their possible myriad interactions. This “menu” of behaviours drove the architecture of the model as it was designed to allow most of these stories to be explicitly represented. When the simulation is run, the local conditions of each agent separately define the context of that agent whilst also allowing the complex mixing of many different social and cognitive processes.

To fill in some of the cognitive and contextual “glue”, evidence from many different sources has been included to motivate the assumptions and mechanisms of the model. Thus it is difficult to identify discrete “sub-models” in this. However, a post-hoc analysis of the structure that emerged suggests the following could be considered as sub-models:

1. The main social unit is the household, a collection of individuals living within the same house. People who partner may form a new household, or people moving from outside the area may also do so. Many social processes occur within the household and others occur preferentially between members of the same household. Households occupy a place on the available square 2D grid.

2. Basic demographic processes specify how people enter the model (though moving into it from the UK or abroad), are born to partners, age, leave home, partner, separate, and die. These are based on some available statistics as to the probability of these events, depending upon whether the immediate situation of the agent makes these plausible (e.g. no one gives birth without a partner, nobody can separate until partnered etc.). This demographic model includes a 5-category social class model, with statistics from class mobility determining when and where people move class.

3. To this basic demographic is added a number of activities. These are currently schools, places of work, activity type1 and activity type2 (these may be thought of as to correspond to things like: places of worship, sports clubs etc.). Agents between the age of 4 and 18 attend school; those 18-65 can go to a place of work, and join (or leave) activities. The activities are recorded as ‘place-holders’ on the grid of households and take up a location but they have no characteristics except their current membership. All children are members of the nearest school; if in work adults are members of a random place of work; with a certain probability adults join an activity and, if so join the one whose other members are (on average) most similar to themselves.

4. A dynamic social network develops between agents. Each link represents a relationship that would allow for a conversation about politics and voting should the participants be up for this. The links are typed and the types are: partner, household, neighbourhood, work, school, activity1 and activity2. There are several different ways that a new link can form: all people in the same household are linked with a household link, there is a chance that people in neighbouring households might link with a neighbouring link, people who go to the same school or parents of children who go to the same school might link with a school link; people who are members of the same activity might form a link. Further for each of these link types there is a chance of making a link with someone linked to someone an individual is linked to (“friend of a friend”). Links can be dropped under certain circumstances and with certain situations (e.g. if one moves, most of the neighbourhood links are lost).

5. Agents can have different levels of political interest (from lowest to highest): not noticing politics; noticing politics; taking a political view on issues; interested in politics, involved in politics. They also have other associated attributes, such as (possibly) a: party political leaning (the party they would vote for if they did), a sense of civic duty to vote, a generalised habit to vote, a party identification, and a memory of whether past voting/not brought about their desired outcome.

6. A process of social influence occurs over this social network in the form of discrete (as opposed to continuous) political discussions. A political discussion occurs if: (a) there is a link between the two (b) the talker is at least interested in politics (has at least a view on politics) and (c) the receiver at least notices political discussions (there is a lower level of awareness that occurs in the home and elsewhere to get people up to the level of noticing political discussions).

7. These political discussions have several possible effects (when taken in aggregate): they may increase the level of political interest of the listener, they may help impact a sense of civic duty and they may help convince the listener to adopt a political leaning. There are some slow processes whereby these may be forgotten over time.

8. When an election occurs, each individual goes through a process which determines whether they vote or not: (1) if they have a sense of civic duty, or general voting habit (2) rational calculations such as whether the balance of past voting experiences was positive and whether they have a strong party identification (3) (if this occurs) political parties may mobilise some who have leanings towards them but were not intending to vote (4) positive intentions to vote may be confounded by factors such as: have a very young baby, having just moved, having just been made unemployed or being to ill to vote, (5) finally those going to vote may “drag” others to come with them and vote, especially partners or family.

9. Voting statistics are then recorded, with agents remembering where and when they voted, with the election result being decided by the majority vote within the model (although an option is that it could be imposed from outside).

The above are not the full details but a summary of their main features. Generally micro-causation in the model happens down the order above (from first to later), but there are some weaker and slower feedbacks that occur back up, for example the outcome of an election effects agents' perceptions of the experience of voting (whether voting resulted in the party they wanted); the characteristics of agents (including party leaning) may affect which activity they join, their friends and who they choose as a partner; and (most importantly) political discussions affect the level of interest of agents.

Emergence

Clearly in such a complicated model it is not possible to make an easy and clean distinction between results that emerge and those that are programmed into the model. Indeed, the model was designed with a view to integrate available evidence rather than produce or demonstrate emergent effects (or to be predictable). However it is not the case that all outcomes from the model are straightforwardly forced by the settings and programmed micro-processes, including the following.

- Although the underlying demographic model is fairly predictable in its unfolding, which partnerships are formed affect which new households with children are created (that do not result from people moving into the district from outside), so the developing social network affects the demographics a little.
- The patterning of households within the 2D space has certain self-organising features. Households have a tendency to move to districts where surrounding households will have some similar agents to themselves, resulting in some weak clustering. The positioning of schools also has an effect as children will go to the nearest school, and links may be formed between parents of children at the same school.
- Agents will tend to choose to participate in (voluntary) activities whose other members are (on average) most similar to themselves, so that these activities tend to act to promote clustering of similar individuals, regardless of location.
- Depending on the network structure, clusters of agents will tend to reinforce patterns of interest/lack of interest in politics. This may reinforce or act against tendencies that might already exist within households of different kinds within the simulations (which will for the reasons above tend to cluster together in terms of location and activity membership etc.).

The initialisation of the model (see below) has a complicated but predictable effect on the model, in that the kinds of household the model is seeded with will affect the tendencies that follow. Thus in the data set that these are selected (at random) from those from "invisible minorities" (Irish etc.) tend to be more politically involved and have a higher sense of civic duty than the native majority population, so if the model is selected to have more of this kind one will find a higher level of turnout.

The impact of many of the parameters is straightforward, for example: increasing the probability of holding a conversation increases the general level of political interest and hence the turnout; increasing the forgetting rate (the "forget-mult" parameter) means that people do not recall so many positive political messages and hence the level of interest in politics falls quicker. The immediate effect of mobilisation is fairly straightforward and the more people are mobilised the more vote – but how this effects

the longer term is less obvious in that it seems to have greatest impact upon the levels of civic duty and general habit, than (for example) in terms of a cascade effect in bringing yet others out to vote.

Adaptation

Agents generally do not seek to increase or optimise any measure of success nor do they reproduce behaviours that they perceive as successful. The exceptions are: (a) when agents weigh up their past experiences of voting as one factor in the decision of whether to vote again, (b) when moving to a new location within the model, the choice might be influenced in the sense of seeking a location with neighbours similar to themselves and (c) if choosing to join a type of activity agents will choose the instance of the activity whose membership is, on average, the most similar to themselves.

Objectives

Agents do not aim to meet any objective.

Learning

Agents do learn, adapting their traits over time depending on their circumstances and history.

- *Level of interest in politics*: this is influenced by many factors, including: amount of political discussion in the parental home, whether they have had a post-18 education, and the level of experienced political discussion once an agent has left home (this needs to be higher than that within the home for the same effect). However level of experienced political discussion only has this effect once an agent gets to the level of noticing politics (or above) which may be triggered by a certain level of discussion in the home, or a much higher level outside.

- *Social network*: agents develop their social network in a number of ways over time: (a) they are automatically linked to other members of the same household, (b) they connect with a probability to those at the same school (or other parents with children at the same school), activity, workplace or immediate neighbours (but preferentially to those more similar to themselves) and (c) for each kind of link (neighbourhood, school, activity1, activity2, workplace) they can make a link to some of those linked to those they are linked to (so called 'friend of a friend'). There is a fixed probability of dropping links at each time click, also if an agent moves they are almost certain to lose existing school, neighbourhood and household links (though there is a small probability of retaining them).

- *Political leaning*: If agents are sufficiently interested in politics, then they can be persuaded to adopt a political leaning in the following conditions: (a) in the home adopt the party of the most politically interested parent, or if both equally interested the party if they agree on this (b) outside the home change from grey (no party) to the most frequently mentioned party in political discussions it has heard, depending on the proportion of discussions for the most mentioned party and its current level of political interest (c) if the political interest level of the agent falls to below 'noticing politics' then they lose their political leaning.

- *Whether they feel a sense of civic duty to vote*: political conversations that are conducted by an agent with civic duty can impart civic duty to another agent.

- *Whether they have picked up the simple habit of voting*: people acquire a habit of voting when they have voted in 3 consecutive elections. If they fail to vote in 2 consecutive elections they lose this habit.
- *Whether they have developed an identification with a particular political party*: if agents have voted in the previous 3 elections for the same party, they acquire an identification with that party. If their politics ever drops to grey (no party) they lose this.
- (During the short run-up to an election) *The level of intention to vote*: at the start of the (short) campaign this will be set for each agent according to a number of factors (whether they have civic duty, have developed a voting habit, have the highest level of interest in politics, have a strong party identification and are statisfied with past voting outcomes, are a loyal supporter and are statisfied with past voting outcomes). As the short campiagn develops conversations might also have the effect of increasing this intention to vote (Depending on the level of intention in both agents).

Prediction

Agents do not do any prediction in this model. In particular, in this version of the model, there is no tactical voting, nor expectations about whether it is worth voting based on predicted outcome.

Sensing

This is a social model, so that agents primarily sense other agents in three ways: (a) through their current links to other agents, (b) through indirect links to other agents, e.g. by being members of the same activity, having kids at the same school or being in neighbouring cells (c) through political discussions over the direct links. Thus all sensing is local in the sense of their links, memberships or neighbourhood (except that agents are aware of the result of elections).

Interaction

Agents interact with each other by having political "conversations", which may influence the recipient. Each "conversation" carries messages of political leaning and civic duty (depending on the characteristics of the converser). These are not strictly conversations since each one is one way, but over time these may go both ways between agents, reinforcing existing characteristics of leaning, political interest and sense of civic duty. If an agent moves location, it will bring its partner and children with it (as well as possibly orphaned children in the household). Agents form sexual partnerships, selecting from those in their social network, and can only have children when within such a partnership. Partnerships dissolve with a low random probability in which case one partner will move out leaving any children behind.

Stochasticity

Many processes in the model have a stochastic element in them once the conditions for their occurrence are locally met in an agent. This includes the processes of: moving location, emigrating, immigrating, getting a job, losing a job, making new social links or losing them, joining an activity or leaving one, having a political conversation, acquiring civic duty as a result of a conversation, dragging others to go and vote if they are going, and mobilising voters. Other process have a probability of occurring but with the

probability varying on the basis of some statistics, including: birth, death, moving out of the parental home, becoming ill, and children changing class later in life from that they were born with (which also depends on having a post-18 education).

The processes that determine the probability of someone voting are deterministic but somewhat complicated (see 8 in the section on design principles and 14 under the section on scheduling). Many circumstances, such as having a sense of civic duty or being politically involved force a probability 1 of voting (unless a confounding factor intervenes).

Processes that are entirely deterministic include: going to school or leaving it, retiring from work, the election result, changes in the habit of voting, or political identification.

A major stochastic impact on the model is in the initialisation of the households at the start of the simulation and the choice of new households that enter during the simulation due to immigration. In these processes entire households are selected at random from re-mixed sample of households from the 1992 wave of the BHPS. The "re-mixing" is done to achieve the user defined proportion of majority population as well as to ensure that out-of-UK immigration is selected from those recorded as immigrants in the BHPS sample. Thus the mix of initial households in each run of the simulation will be somewhat different, but on the whole, the balance of household characteristics will be representative for simulations with larger populations albeit with some stochastic variation.

Collectives

Some of the agent characteristics do influence how the agents make links and move. Which locations a household moves to is influenced by a bias towards moving next to households with similar characteristics; which instance of a kind of activity 1/2 are joined will be those whose existing members have (on average) the most similar characteristics as themselves; which person they make links with via an activity will be biased by a similar homophily formula. Thus over time agents will tend to have more links with those similar to themselves. However due to the presence of much stochasticity in the model this does not produce pronounced segregation, but rather a "softer" bias in terms of social links. The characteristics that are involved are: age, ethnicity, class and political leaning. At the moment there is a single dissimilarity measure used between two agents regardless of the context (in future versions this will be changed so that there are different measure for different circumstances, so (for example) a weaker one at work than for choosing which instance of an activity to join).

Political parties are not currently represented, except implicitly in terms of the mobilisation process. Individuals influence each other individually and not collectively in this model.

Observation

Many different statistics are collected from the simulation. Broadly the more complex a simulation, the more different aspects need to be validated in order to have any confidence that the model represents what one intends it to. Following the process of cross-validation [5] broad evidence and statistics are used to inform the specification micro-level agent rules but then the results coming out of the model also checked, both statistically and in broader qualitative terms. We will not describe all these here. More details can be found in the documents archived with [3]. These include output statistics, graphs, histograms, a visualisation of the world with the social netoworks and agents shown, and there is a trace, where the events that occur to a randomly chosen agent are

logged. When this agent dies a new born agent is chosen and logged. This is to give a feel for the sort of life trajectories agents are going through.

Details

Initialization

The grid is initialised in the following manner:

- The grid dimensions are set by the programmer
- Set proportions of the grid are occupied with schools, work places, activity1 and activity2 (with a minimum of one each)
- A given proportion of patches that are left are populated with new households. These are selected as a complete household from a large sample of taken at random from the 1992 wave of the British Household Panel Survey (BHPS) [1], but 'remixed' to a set degree of majority population (by splitting the original file into majority/non-majority households and then probabilistically choosing at random from each part according to parameter settings). Some details about households (e.g. which child in a household belongs to which parent) have to be inferred from the data as this is not always unambiguous. Some initial agent characteristics are set using proxies from the data, e.g. civic duty is set for agents who are recorded as being a member of certain kinds of organisation
- Links to household members and some random neighbours are made
- To give the households an initial network the procedure to develop other network links is done 10 times for each household.
- Appropriate activities are joined depending on those in the BHPS data. Thus the exact composition of the grid varies in each run but are drawn from the same sample, so in a sufficiently large initial set of households (determined by the size of the grid and how much is left empty) one gets a similar mixture each time. Various other things are initialised including: shapes and colours for main display, election dates, and party labels.

Input Data

There are two sets of data that are used in the model:

- A sample of the 1992 wave of the BHPS data as described above. This file cannot be distributed due to UK Data Archive restrictions and it will be soon available on their site. In its stead we are distributing the model with synthetic data which does not relate to any real individuals but has some of the same characteristics as the original file [3].
- Various statistics concerning the underlying demographics, such as birth rate (depending on the age of parent), death probability (each age), probability of males and females leaving home. At the moment these are statistics from only roughly the appropriate time.

Submodels

It is important to understand that this is *not* a simulation with free-parameters that are conditioned on some "in-sample" data. It does have a lot of parameters, but these are set (or could be set) from empirical data. The model is then run "as is" and can be compared with available data — to see how and where it matches this and when it does not. Thus (unlike many models) it is not an attempt to 'fit' any data, but rather is a computational description to enable the 'detangling' and critique of various explanations of observed social behaviour.

Some of the principal parameters that have real referents (that is, in principle they could be determined from empirical data), include the following:

- drop-friend-prob: the probability a link is dropped in a year
- drop-activity-prob: the probability an activity membership (not work or school) is dropped each year
- prob-partner: the probability of forming a sexual partnership if single per year
- prob-move-near: when a household moves this is the probability it moves to the nearest empty patch rather than to a patch with similar neighbours to itself
- immigration-rate: percentage of population that immigrates from outside the UK into the model (and hence is randomly selected from the immigrants section of the BHPS file)
- int-immigration-rate: percentage of population that immigrates from inside the UK into the model (and hence is randomly selected from the re-mixed version of the BHPS file)
- emigration-rate: the rate (per year) that households leave the model
- dissim-of-empty: when judging if a neighbourhood contains similar households to self, this is how dissimilar an empty space is (thus a low value of this results in housholds seeking to move near empty spaces, a high value to avoid empty spaces)
- election-mobilisation-rate: the percentage of its supporters who are not intending to vote that a party tries to get to vote
- start-mobilisation: when party mobilisation starts
- end-mobilisation: when party mobilisation stops

The following allow the turning on and off of various processes or structures and thus allows the comparison of the simulation behaviour with and without them.

- household-drag?: whether agents attempt to drag others to vote
- rand-convs?: if on means that political conversations happen at random and are not constrained by the social network
- p2p-influence?: switches whether the specific influence between discussants during the election period on their intention to vote can occur
- no-rat-voting?: turns off the calculative (or "rational") aspects of the decision whether to vote
- greys-vote?: whether those with no political inclination can vote (if they do they do so randomly)

- mob-once-ph?: whether mobilisation conversations only occur once to each household
- fof?: switches the friend-of-a-friend social link creation mechanism

Some of the other parameters can be used to implicitly switch processes on and off:

- influence rate: setting this to zero switches off all political conversation (apart from mobilisation conversations)
- prob-contacted: setting this to zero switches off mobilisation during elections
- major-election-period and minor-election-period: setting these to zero switches off elections
- immigration-rate and int-immigration-rate: setting these to zero switches off any incomers to model (warning may critically affect longer-term population levels)
- emmigration-rate: setting this to zero switches off any emigration model (warning may critically affect longer-term population levels)
- birth-mult: setting this to zero switches off any births (warning may critically affect longer-term population levels)
- death-mult: setting this to zero switches off any deaths (warning may critically affect longer-term population levels)
- prob-partner: setting this to zero switches off any partnering after initialisation (warning may critically affect longer-term population levels)
- separate-prob: setting this to zero switches off any separation of partners (warning may critically affect longer-term population levels)
- forget-mult: setting this to zero switches off any forgetting of conversations etc. by agents (warning will cause model to slow down as agent accumulate huge lists of memories)
- move-prob-mult: setting this to zero switches off any moving within model

The following affect the initialisation of the simulation.

- density: the initial density of households in the spaces left for them after schools etc. have been allocated
- majority-prop: the proportion of the initial population from the majority group
- init-move-prob: how many times households are moved in the initialisation (this produces a slightly more realistic starting point for the model with weak clustering)

The following control how the simulation run occurs and what data is output.

- start-date: year simulation starts
- end-date: year simulation finishes
- ticks-per-year: how many simulation ticks are in each year and probabilities throughout the simulation are adjusted so that roughly the same will happen with different settings of this, so as to enable fast debugging runs with 1 tick per year before slower ones with 12. However there will be subtle differences in model behaviour for different settings of this.

- to-file?: switches whether simulation saves statistics to the file given in "output-filename"
- when-calc-data?: determines when the simulation saves statistics and/or network data (1=every tick, 2=every two ticks, etc.)
- sna-out?: switches whether the simulation outputs the current social network (one file each time is does this!)

The following are scaling parameters.

- birth-mult: a scaling parameter that changes the birth rates uniformly
- death-mult: a scaling parameter that changes the death rates uniformly
- move-prob-mult: a scaling parameter that changes the probability of moving
- influence-rate: a scaling parameter determining the maximum number of chances to influence others each agent has each year (this will be realised by very few agents if any, but will have the effect of scaling the number of discussions agents who are politically interested agents have)
- forget-mult: a scaling parameter that changes the rate of forgetting

References

1. British Household Panel Study (BHPS);. https://www.iser.essex.ac.uk/bhps.

2. Grimm V, Berger U, Bastiansen F, Eliassen S, Ginot V, Giske J, et al. A standard protocol for describing individual-based and agent-based models. Ecological modelling. 2006;198(1):115–126.

3. Edmonds B, Lessard-Phillips L, Fieldhouse E. A Complex Model of Voter Turnout (Version 1); 2014. CoMSES Computational Model Library. https://www.openabm.org/model/4368/version/1.

4. Edmonds B, Moss S. From KISS to KIDS–an 'anti-simplistic' modelling approach. Springer; 2005.

5. Moss S, Edmonds B. Sociology and Simulation: Statistical and Qualitative Cross-Validation1. American journal of sociology. 2005;110(4):1095–1131.
