# Supplementary material for: Correction: Correction: Staged Models for Interdisciplinary Research
Source: PLoS One. 2016 Oct 18;11(10):e0165250. doi: 10.1371/journal.pone.0165250 (PMC5068733; doi:10.1371/journal.pone.0165250)
Supplement: S1 File — (PDF) [file pone.0165250.s001.pdf]

PLOS | ONE

CORRECTION

Correction: Staged Models for Interdisciplinary Research

The *PLOS ONE* Staff

Figs 1 and 2 are incorrectly swapped and the legend for Fig 4 is incorrect. The corrected figures and captions are provided here.

CrossMark
click for updates

OPEN ACCESS

Citation: The *PLOS ONE* Staff (2016) Correction: Staged Models for Interdisciplinary Research. PLoS ONE 11(8): e0162151. doi:10.1371/journal.pone.0162151

Published: August 25, 2016

Copyright: © 2016 The PLOS ONE Staff. This is an open access article distributed under the terms of the Creative Commons Attribution License, which permits unrestricted use, distribution, and reproduction in any medium, provided the original author and source are credited.

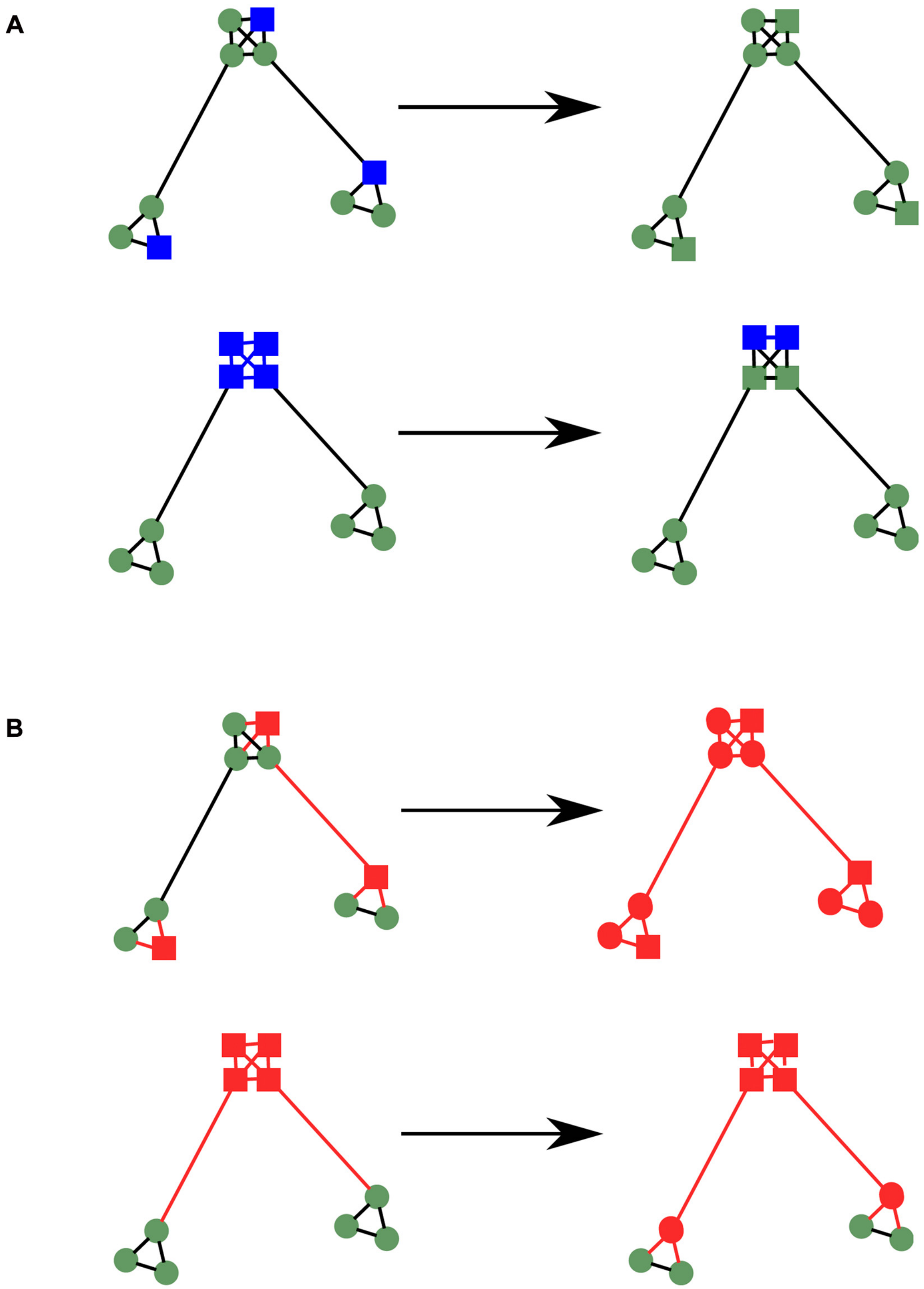

Fig 1. From a single to a multi-stage abstraction process.

doi:10.1371/journal.pone.0162151.g001

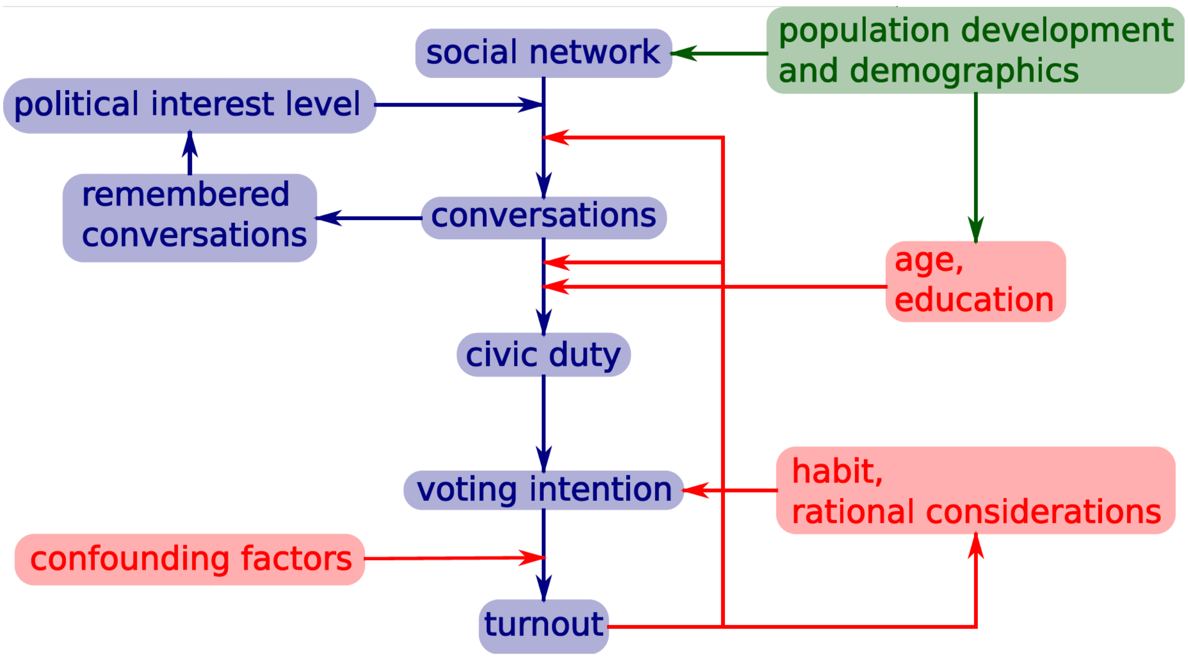

Fig 2. Diagrammatic representation of the full model processes. The main pathway is shown in blue, with additional factors in red, and development of the agent population in green.

doi:10.1371/journal.pone.0162151.g002

A

B

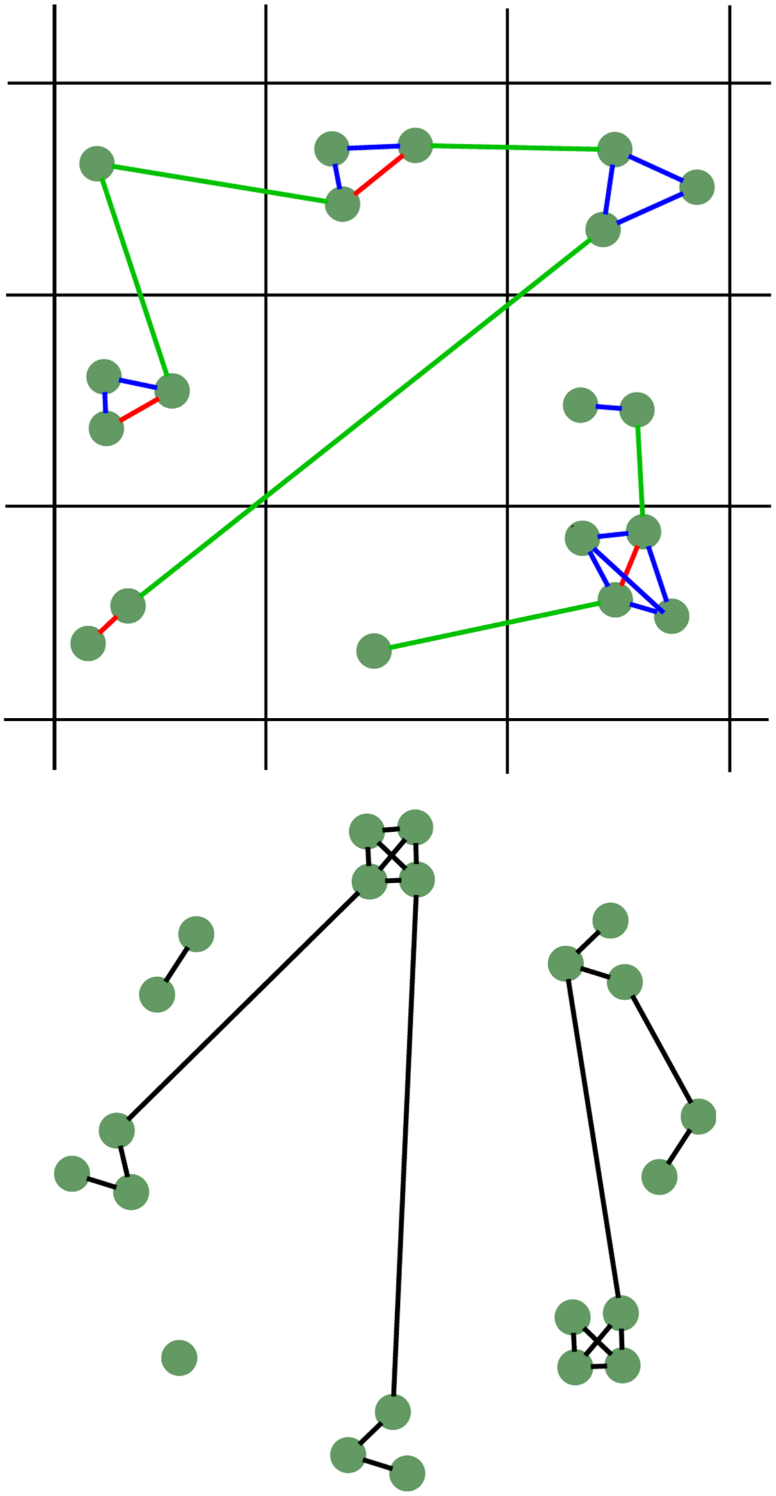

Fig 4. Schematic comparison between the full model network (A, above) and the synthetic network (network CN, B below). Agents are displayed as green circles. Lines connecting agents represent social links. In the full model red lines represent partners, blue lines represent families and green lines represent other kinds of relationships.

doi:10.1371/journal.pone.0162151.g003

Reference

1. Lafuerza LF, Dyson L, Edmonds B, McKane AJ (2016) Staged Models for Interdisciplinary Research. PLoS ONE 11(6): e0157261. doi: 10.1371/journal.pone.0157261 PMID: 27362836
